# Purple potato extract modulates fat metabolizing genes expression, prevents oxidative stress, hepatic steatosis, and attenuates high-fat diet-induced obesity in male rats

**DOI:** 10.1371/journal.pone.0318162

**Published:** 2025-04-01

**Authors:** Ishrat Jahan, Asif Ul Haque Shuvo, Mirza Alimullah, A. S. M. Nafiur Rahman, Shahnaz Siddiqua, Shatil Rafia, Ferdous Khan, Khondoker Shahin Ahmed, Hemayet Hossain, Kazi Akramuddaula, Md Ashraful Alam, Nusrat Subhan

**Affiliations:** 1 Department of Pharmaceutical Sciences, North South University, Dhaka, Bangladesh; 2 Department of Pharmacy, East West University, Dhaka, Bangladesh; 3 Chemical Research Division, BCSIR Laboratories, Dhaka, Bangladesh Council of Scientific and Industrial Research (BCSIR), Dhaka, Bangladesh; 4 Pharmacy Discipline, Khulna University, Khulna, Bangladesh; Georgia State University, UNITED STATES OF AMERICA

## Abstract

**Objective:**

In this investigation, the significance of purple potato (*Solanum tuberosum L*.) extract treatment was assessed against oxidative stress and fat metabolizing transcription factors in the liver of high-fat (HF) diet-fed rats.

**Methods:**

Wistar (male) rats were arranged into several groups and provided with a control and HF diet along with the purple potato extract. Body weights, oral glucose tolerance test (OGTT), insulin, plasma lipids, and oxidative stress-related indicators were analyzed in plasma and tissue samples. Additionally, real-time PCR was performed to evaluate the gene expression for oxidative stress and fat metabolism in the liver. Histological staining was also performed on pancreatic and hepatic tissues.

**Results:**

Purple potato extract lowered body weights and improved glucose utilization in the OGTT test in HF diet-fed rats. Purple potato extract also suppressed HF-diet-induced oxidative stress in plasma and hepatic tissues. Purple potato extract also restored the Nrf-2 expression in the liver, followed by the improved expression of HO-1, HO-2, and other antioxidant genes in HF diet-fed rats. In addition, genes involved in lipid metabolism were also positively modulated due to purple potato extract treatment. Furthermore, histological examination revealed the reduction of lipid accumulation and amelioration of inflammation due to the consumption of purple potato extract.

**Conclusion:**

This investigation revealed that antioxidant-rich purple potato extract can modulate the antioxidant and fat metabolizing genes expression, ameliorated oxidative stress and glucose intolerance as well as lowered blood lipids in male rats.

## Introduction

Obesity is becoming prevalent in recent world and poses a substantial public health risk. According to the World Health Organization, the incidence of obesity has doubled since 1980, with around 39% of people globally being overweight and 20% being obese in 2020 [1]. White adipose tissue (WAT) growth is a hallmark of obesity, which mostly leads to abdominal fat deposition [2]. Due to a few underlying pathophysiological mechanisms, obesity has been linked to a variety of issues. Numerous biological systems might experience difficulties due to obesity, including metabolic syndrome, type 2 diabetes mellitus (T2DM), non-alcoholic steatohepatitis (NASH), and cardiovascular issues [3]. Obesity has a very complex and convoluted pathogenesis, however, the reasons which may develop obesity are daily high-fat (HF) containing cafeteria type meal and a lack of regular movement. A high-fat (HF) diet promotes the hypertrophy of white adipose tissue and the production of pro-inflammatory adipokines [4]. An HF diet causes fat accumulation in hepatic tissues, steatosis, and the formation of non-alcoholic fatty liver disease (NAFLD) [5]. NAFLD is a series of hepatic disorders that develop from fatty liver to hepatocytes degradation, and fibrosis [6]. Excess triacylglycerols in the liver’s cells can get preserved in the form of tiny droplets of lipid in hepatocytes. Subsequently, modern affluent diets increase the deposition of excess fat in hepatocytes by giving more dietary triacylglycerols, fatty acid, and glucose [7]. Steatosis and insulin resistance can induce and exacerbate one another, resulting in a recursive process of metabolic failure [8].

The transition from hepatic steatosis to steatohepatitis involves oxidative stress, which is a major part in the development of steatohepatitis [9]. Fatty liver is particularly prone to aggravate oxidative damage [10]. Though pro-oxidants are necessary for the killing of harmful pathogens, an excess of pro-oxidants causes lipid peroxidation. Biochemical changes in genetic materials have the potential to become deleterious to cells or can trigger a host-immune response, resulting in inflammation, formation of collagen, and the development of disease. Mechanisms that promote pro-oxidant formation comprise mitochondrial malfunction (NADPH oxidase and electron transport chain leakage) and activation of liver cytochrome P450 (microsomal, peroxisomal CYP2E1) due to the overburdening of the fatty acid oxidative pathway [11]. It has been shown that insulin resistance may promote oxidative stress by increasing microsomal lipid peroxidases and reducing beta–oxidation pathway in mitochondria [12].

Weight loss strategy involves low-calorie diet intake, exercise, lifestyle modification, and medication. Several drugs are under investigations, and some are also used clinically recently such as metformin, orlistat etc. [13,14]. Orlistat is a gastric and pancreatic lipase inhibitor which prevents fat digestion and absorption from the gut [15]. Recent investigation showed that orlistat administration prevented liver damage and restored the antioxidant defense mechanism by activating the Nrf-2 mediated pathway [16]. Orlistat treatment also lowered the total cholesterol and triglyceride levels, insulin resistance, and NASH in HF diet-fed rats [13,16]. In recent years, many investigations have tried to identify an association between antioxidant compounds and oxidative stress caused by obesity [17]. Antioxidants are substances that reduce reactive oxygen species (ROS) levels and can alter the pathways that keep glucose, lipids, and amino acids in a stable balance, thereby inhibiting the inflammatory response [18]. Flavonoids are polyphenolic antioxidant molecules that, in addition to being metal chelators and ROS scavengers, have the potential to provide twofold protection against oxidative stress [19]. Polyphenols in the diet can significantly reduce the chances of acquiring a variety of disorders, including cancer, cardiovascular disease (CVD), diabetes, inflammatory diseases, and neurological disorders. Polyphenols are capable of neutralizing the ROS or decrease cellular oxidative stress (OS), to prevent oxidation to macromolecules such as lipids, proteins, and DNA, which in turn reduce tissue inflammation [20].

Potato (*Solanum tuberosum L.)* is one of the important food crops, alongside grain, rice, and maize. Potatoes may offer glucose, high-quality protein, and vitamin C to individuals; potato starch can also be utilized for industrial processes [21]. Various varieties of potatoes are growing around the world. The purple potato is one of the cultivars among the potato varieties. Significant levels of phenolic, flavonoid, and anthocyanin chemicals were discovered in the purple potato [22]. The anthocyanin concentration in potato stem tubers is responsible for the purple color. The potato anthocyanins exhibit considerable anti-oxidative activity [23]. The relationship between anthocyanin concentration and anti-oxidative activity is beneficial, and it leads to several significant pharmacological benefits. It is well established that anthocyanins have strong anti-diabetic effects [24]. Proteinase inhibitor II, a component of purple potatoes, catalyzes the release of cholecystokinin (CCK), delaying stomach emptying in people [25]. In addition, potato anthocyanins may improve colonic environments, improve the epithelial differentiation in the gut, and facilitate barrier functions via AMPK activation [26]. Purple potato extract exhibited hepatoprotective effects, suppressing lipid peroxidation and inflammation in the liver of D-galactosamine-treated rats [27]. Considering these above-mentioned facts, this investigation was undertaken to demonstrate the benefits of purple potato extract in lowering the adipose tissues, plasma cholesterol, and triglyceride levels in HF diet-fed rats. Moreover, the preventive effect of purple potato extract against oxidative damage, and on NAFLD in rats fed an HF diet were also evaluated.

## Materials and methods

### Chemicals, reagents and kits

From Sigma-Aldrich (St. Louis, MO, USA), gallic acid, kaempferol,3,4-dihydroxy-benzoic acid, quercetin, vanillic acid, and catechin hydrate, rosmarinic acid were purchased. Methanol, ethanol, acetic acid, and acetonitrile were acquired from Merck (Darmstadt, Germany); all are HPLC grade. We received kits to measure the enzymes alanine transaminase (ALT) (Ref. No.: 41255), aspartate transaminase (AST) (Ref. No.: 41256), and alkaline phosphatase (ALP) (Ref. No.: 41257) from Clinichem Limited (Budapest, Hungary). The total cholesterol (Ref. No.: 41263) and triglyceride (Ref. No.: 41266) assay kits were also obtained from Clinichem Limited (Budapest, Hungary). For the oxidative stress markers and picrosirius red staining assays, samples and all other reagents were purchased from Merck (Darmstadt, Germany) and Sigma-Aldrich (St. Louis, USA). The thiobarbituric acid was collected from Sigma Chemical Company (St. Louis, Missouri, USA). Reduced glutathione (GSH) was purchased from J.T. Baker (USA). From SR Group (Delhi, India), SOD standards and other test components have been purchased. The SYBRTM Green PCR Master Mix, RevertAid First Strand cDNA Synthesis Kit (Catalog number: K1621), and GeneJET RNA Purification Kit were supplied by Thermo Fisher Scientific Inc. (Waltham, Massachusetts, United States of America). All additional substances and chemicals have been classified as analytical-grade reagents.

### Purple potato (*Solanum tuberosum L.*) extract preparation

Purple potatoes (*Solanum tuberosum L.)* were collected from a regional market in Dhaka district, Bangladesh (in April 2020) and identified at the Bangladesh National Herbarium, Mirpur, Dhaka. The purple potato voucher specimen has been stored for clarification purposes, and a catalog number was provided, which is DACB No: 66756. Experimental research and field studies on plants (either cultivated or wild), including the collection of plant material, comply with relevant institutional, national, and international guidelines and legislation. These potatoes were then carefully stored in a storage room. The collected potatoes were cleaned to remove unwanted substances and dirt before drying in the sunlight. These chopped potato cubes were steamed in a steam machine for 15 minutes. It was then mashed with a hand mixer. Potatoes were dried and ground again. After drying, the purple potato was ground into a fine powder using a grinder machine. To yield the extract, this fine powder (100 g) was soaked in ethanol (95%) for several days in a closed glass jar. After maceration for extraction, the mixture was then filtered carefully to remove the debris. A concentrated purple extract was procured after evaporating the ethanol in a rotary evaporator. Along with the diet, this crude extract was used as a treatment in rats. Throughout the experiment, the extract was kept in an airtight container in a cool, dry, and dark place.

### HPLC detection and quantification of polyphenolic compounds

In a 25 mL volumetric flask, 16 phenolic compounds were dissolved in methanol to create standard conventional solutions. The stock solutions’ concentrations ranged from 4.0 to 50 μg/ml. In order to generate the operational standard solutions, the necessary volumes of each stock solution were incorporated in tandem and periodically diluted. Every solution was kept in the refrigerator for further analysis. High-performance liquid chromatography-diode array detection (HPLC-DAD) analysis was used to identify and measure specific polyphenolic chemicals present in purple potato extracts, as stated in a previous report [28] with some modifications. HPLC analysis was successfully carried out on a Shimadzu (LC-20A, Japan) equipped with a column oven (CTO- 20A), an autosampler (SIL-20A HT), an array of photodiode detectors (SPD-M20A), and a binary solvent delivery pump (LC-20AT). For isolation at 33°C, a Luna C18 (5 m) Phenomenex column (4.6 x 250 mm) was applied. Solvent A (1% acetic acid in acetonitrile) and Solvent B (1% acetic acid in water) constituted the mobile phase. The entire quantity injected was 20 μL, and the flow rate was 0.5 mL/min. The steep gradient elution scheme was created to separate the chemicals in the sample to be analyzed. The column was washed with the original 5% A mobile phase to eliminate any partially bound chemicals. The compounds having a medium affinity for the stationary phase then began to elute as the mobile phase was gradually altered to 25% A.

### Animals

The Department of Pharmaceutical Sciences at North South University supplied 30 male Wistar rats (10-12 weeks old, 185-215g) through the animal breeding division. Every rat was kept in separate cages, having food and water readily accessible throughout the times. The surroundings were kept at a constant temperature of 23°C, humidity of 52-55%, and maintained a 12-hour light/dark cycle. The diet was divided into two groups: the high-fat (HF) diet and the control diet. The HF diet included condensed milk, sugar, beef tallow, and chow food. The HF diet composition contains around 49% fat, 14% protein, and 37% carbs. The control diet consisted of corn starch powder, chow food powder, vitamins, salt, and water, with a composition of around 60% carbohydrate, 15% fat, and 25% protein. The composition of the diet can be found in Table 1. Energy calorie intake was estimated from the following values in kilojoules per gram: sucrose, 16.5; corn starch, 15.94; condensed milk, 13.80; beef tallow, 37.70; and powdered rat food, 13.80. The energy densities of the control and HF diets were 11.23 kJ/g and 17.83 kJ/g of food, respectively [29]. Gas chromatography analysis showed that the control food contained palmitic acid, oleic acid, and linoleic acid as the major fatty acids; while the HF diet contained a high amount of palmitic acid, oleic acid, and stearic acid compared to the control food (S1 File), (S1 and S2 Figs), (S1 and S2 Tables) [30].

**Table 1:** Composition of Control diet and High fat diet used (approximately for 100 g) in this study.

High Fat Diet Composition (100 g)	Percentage (%)	Control Diet Composition (100 g)	Percentage (%)
Powdered chow food	15.5	Corn Starch	60
Sugar	17.5	Powdered Chow Food	15.5
Beef tallow (fat)	20.0	Vitamin B complex	0.1
Condensed milk	39.5	Salt	2.5
Vitamin B complex	0.1	Water	q.s.t
Salt	2.5		
Water	q.s.t		

The control diet contains approximately 25% proteins, 60% carbohydrates and 15% fat in terms of the caloric content. On the other hand, the high-fat (HF) diet was composed of normal food, beef tallow, sugar and condensed milk and contained approximately 14% proteins, 37% carbohydrates and 49% fat in terms of the caloric content. q. s. t- quantity sufficient to.

### Study design

All experimental protocols were authorized by North South University’s Ethical Committee (IACUC-ID: 2022/OR-NSU/IACUC/0302). We are also confirming that the study was conducted in accordance with ARRIVE guidelines. Rats were divided into five experimental groups (N = 6). Animals in group-I, which served as the control, were fed a control diet and water. They also received the vehicle every day through oral gavage. Group-II rats were given the HF-diet and water. These rats also received the vehicle every day through oral gavage. Group-III rats were given a control diet and water, together with purple potato extract at a dose of 100 mg/kg daily by oral gavage for 8 weeks (control +  purple potato). Rats in group-IV were given both the HF-diet, water, and purple potato extract (HF +  purple potato) at a dose of 100 mg/kg daily by oral gavage for 8 weeks. The 8 weeks’ time was selected based on our earlier investigation which showed that 8 weeks of HF diet feeding in animals is sufficient to develop pathological consequences in experimental animals [30]. A previous report suggests that the daily intake of polyphenolic compounds in humans is approximately one gram per day [31]. Based on this information, we have selected the dose of 100 mg/kg per day in rats. The dose conversion formula between humans and rats reported in previous literature suggests that 100 mg/kg dose in rats is approximately one gram per day in humans [32].

Additionally, Group-V rats received both HF-diet, water, and orlistat at a dose of 10 mg/kg, administered through oral gavage, and mixed in the vehicle. Orlistat is used as a weight loss drug clinically; thus, it is introduced as a positive control. Olive oil was used as a vehicle and the extract-drug-vehicle mixture volume was not more than 0.5 mL. All rats were provided with sufficient drinking water in a standard water bottle.

An oral glucose tolerance test (OGTT) was carried out on all groups of animals prior to the commencement of the experiment as well as at the culmination of the investigation to measure the glucose absorption in the blood due to HF-diet feeding in rats. The body weight, food, and water intake statistics were recorded on a daily basis.

### Animal sacrifice and sample collection

After the 56^th^ day of the experiment, all rats were assessed for body weight and given a high dose of ketamine (90 mg/kg) injection to knock them out before being sacrificed. Blood (5-6 mL) was drawn from the thoracic aorta of the sacrificed animals and preserved in a blood collection tube containing citrate buffer. After 15 minutes of centrifugation at 6500 g, the plasma was separated and kept at -20 °C for future analysis. The internal organs (adipose tissues, pancreas, and liver) were also cut from the animal body, kept in a cold ice bag, and weighed instantly. One part of the liver, pancreas, and adipose tissues from every animal were preserved in a small sample vial containing neutral buffered formalin (pH 7.4) for histopathological investigation (N = 6). A second part of the wet liver tissues was isolated (1 g) in an RNAase-free condition, stored in a microcentrifuge tube, and preserved in a freezer (-80°C) for gene expression analysis. Another part of the liver tissues was stored in a microcentrifuge tube and preserved in a freezer (at -20 °C) for subsequent studies (N = 6).

### Insulin analysis in the plasma

Plasma samples were used to measure the quantitative determination of insulin in rats using an Ultra-sensitive Rat insulin ELISA kit obtained from Crystal Chem Inc. (Downers Grove, Illinois, United States of America, Catalog no. 90060). Rat insulin stock solution and anti-insulin enzyme conjugation reagents were prepared according to the manufacturer’s assay procedure. A standard calibration curve was prepared, and the plasma level of insulin was calculated using this standard curve equation.

### Oral glucose tolerance test

Before the oral glucose tolerance test (OGTT), all test animals were fasted for 12 hours. However, these rats have free access to drink water. Blood was taken from the tail tip minimizing the stressful situations and basal blood glucose levels using a glucometer (Bionim Corporation, Bedford, MA, USA). Following then, an oral dose of 2 grams of glucose per kilogram of body weight prepared in distilled water was supplied to each animal through oral gavage. The blood glucose levels were monitored with the same glucometer at multiple intervals (30, 60, 90, and 120 minutes) following glucose administration.

### Preparation of tissue samples for the assessment of oxidative stress marker

Liver tissues were homogenized in phosphate buffer (pH 7.4) using an ultrasonic homogenizer [33,34]. The cells are disassembled during this process, releasing the proteins and enzymes present inside. A protease inhibitor cocktail (Tocris Bioscience, Bristol, Avon, United Kingdom, Catalogue no. 55005599) was used to inhibit the protein breakdown. This homogenized mixture was then centrifuged at 7000 g for 15 minutes at 4°C to separate the supernatant. Enzymes and proteins that are soluble are found in the supernatant, The supernatant was separated from the tissue debris and preserved in a freezer (at -20 °C) and used for biochemical assays. The protein content was measured using a Bicinconinic acid (BCA) based protein assay kit (Thermo Fisher Scientific, Waltham, USA) (Catalog no.: A55865).

### Assessment of lipid peroxidation

To detect liver lipid peroxidation, reactive thiobarbituric acid substances (TBARS) were quantified by colorimetric method, followed by a previously published technique [35]. Malondialdehyde (MDA), a consequence of lipid peroxidation, is measured by the TBARS assay. To carry out the TBARS experiment, 2 mL of TBA-TCA-HCl reagent (0.37% thiobarbituric acid, 15% TCA, and 0.25 N HCl in a 1:1:1 ratio) are added to 0.1 mL of the tissue homogenate (Tris-HCl buffer, pH 7.5) or plasma. The resulting mixture is put in a sealed Eppendorf tube and heated for approximately fifteen minutes in a hot water bath. The mixture was brought to atmospheric temperature and then centrifuged to separate the pellet from the supernatant. The absorbance of the supernatant was then taken at 535 nm in a UV-spectrophotometer. The MDA standard curve is then used to calculate the MDA concentration. Both nmol/mL and nmol/g tissue units were used to express the MDA concentration.

### Assessment of nitric oxide (NO)

A previously reported test procedure was used to measure nitric oxide (NO) levels. Instead of using 5% 1-naphthyl amine, 0.1% w/v naphthyl ethylene diamine dihydrochloride was used to modify the Griess-Illosvoy reagent [36]. In this process, 0.5 mL of phosphate-buffered saline was added to 2 mL of homogenized tissue and incubated for 150 minutes at 25 °C. The UV-visible spectrophotometer was used to take the absorbance at 540 nm of the final reaction mixture. The NO concentration in unknown samples was measured using a standard curve and represented as nmol/mL or nmol/g of tissue.

### Assay of advanced oxidation protein products (AOPP)

The previously described approach was used to calculate the AOPP level in plasma and tissues [37]. In a test tube, 2.0 mL of plasma or tissue homogenates supernatant was diluted to a 1:5 ratio with phosphate-buffered saline (PBS) solution, followed by adding 0.1 mL of 1.16 M potassium iodide (KI) solution. After 2 minutes, the tubes were filled with 0.2 mL of glacial acetic acid. The absorbance of the resultant combination was measured immediately at 340 nm in comparison to a blank solution (0.1 mL KI +  0.2 mL acetic acid +  2 mL PBS). The standard curve was created using chloramine-T solutions with concentrations ranging from 0 to 100 nM. The concentration of AOPP was calculated using the standard curve equation.

### Determination of antioxidant enzyme activities

The protocol that was outlined in the previous report, was used to determine catalase activity [38]. In this test, one unit of catalase activity was interpreted as an absorbance change of 0.01 units/min. The absorbance changes of the reaction solution in one minute were taken at 240 nm.

The superoxide dismutase (SOD) activity was also assessed in tissue homogenates and plasma samples using a previously reported technique [39,40]. At 480 nm, the variations in absorbance were monitored for 1 min at 15 second intervals. The materials for the control group, which lacked just the enzyme preparation, were used concurrently. One unit of SOD enzyme activity was determined as 50% inhibition of epinephrine auto-oxidation.

The assay of reduced glutathione (GSH) was made following a technique that was explained in previous reports [41,42]. The absorbance of the final reaction mixture was measured at 412 nm using a UV- Spectrophotometer, and the level of GSH was reported as ng/mg of protein.

### Determination of myeloperoxidase (MPO) activity in liver

The previously established di-anisidine-H_2_O_2_-based assay method evaluated MPO activity [43]. This approach was adapted to work with 96-well plates. In each well, 10 microliter plasma sample was combined with H_2_O_2_ (0.15 mM) in potassium phosphate buffer (50 mM) at pH 6.0 and O-dianisidine dihydrochloride (0.53 mM). The absorbance change was measured at 460 nm and reported as MPO activity/mg protein.

### Determination of cholesterol and triglyceride level in plasma

Plasma total cholesterol and triglyceride levels were analyzed by using corresponding assay kits obtained from Clinichem Limited (Budapest, Hungary) following the manufacturer’s assay protocol.

### Estimating the fat metabolizing, inflammation, and oxidative stress regulatory gene expressions

The mRNA was extracted and purified from the liver tissues using the GeneJET RNA Purification Kit from Thermo-Fisher Scientific (Massachusetts, USA). This kit system separates mRNA from other RNA species and impurities using a column-based approach. Applying a NanoDrop 2000 spectrophotometer (Bio-Rad, California, USA), the amount of mRNA was measured in the isolated sample. Maxima SYBR Green qPCR master amalgamates (Thermo Scientific, USA) and a CFX96 C1000 Touch Real-Time PCR (Bio-Rad, USA) were used to construct cDNA for quantitative real-time PCR analysis of transcription factors and enzymes associated with oxidative stress and inflammation. According to the manufacturer’s user’s manual for CFX Manager TM Software (CFX Manager TM Software), the collected information was evaluated. Primer 3, a software program, was used to build the oligonucleotide sequences shown in Table 2. These sequences were then used as forward and reverse primers in quantitative real-time PCR. A 40-cycle polymerase chain reaction (PCR) was conducted at 95 °C for 1 minute, resulting in denaturation at 95 °C for 5 seconds and annealing at 60 °C for 30 seconds, extension at 72 °C for 1 minute, and final extension at 72 °C for 5 minutes. By adjusting it to the expression of the β-actin gene as a control, the transcript level of each target gene was ascertained.

**Table 2:** The forward and reverse sequence of the primer applied in this experiment.

Name of gene	Type	Sequence
Nrf-2 (*nfe2l2*)	Forward	5′-CCC AGCACA TCC AGACAGAC-3′
	Reverse	5′-TATCCAGGGCAAGCGACT C-3′
Heme oxygenase-1 (*hmox1*)	Forward	5′-TGCTCGCATGAACACTCTG-3′
	Reverse	5′-TCCTCTGTCAGCAGTGCCT-3′
Heme oxygenase-2 (*hmox2*)	Forward	5′-CACCACTGCACTTTACTTCA-3′
	Reverse	5′-AGTGCTGGGGAGTTTTAGTG-3′
SOD (*sod1*)	Forward	5′-GCTCTAATCACGACCCACT-3′
	Reverse	5′-CATTCTCCCAGTTGATTACATTC-3
Catalase (*cat*)	Forward	5′-ATTGCCGTCCGATTCTCC-3′
	Reverse	5′-CCAGTTACCATCTTCAGTGTAG-3′
Glutathione peroxidase (*gpx1*)	Forward	5′-GGGCAAAGAAGATTCCAGGTT-3′
	Reverse	5′-GGACGGCTTCATCTTCAGTGA-3′
β-Actin (*actb*)	Forward	5′-GCGAGAAGATGACCCAGATC-3′
	Reverse	5′-GGATAGCACAGCCTGGATAG-3′
SREBP-1c (s*rebf1*)	Forward	5′-GGCATGAAACCTGAAGTGGT-3’
	Reverse	5′-TGCAGGTCAGACACAGGAAG-3’
FAS (*fasn*)	Forward	5’-TCGAGACACATCGTTTGAGC-3’
	Reverse	5’-CTCAAAAAGTGCATCCAGCA-3’
PPAR-γ2 (*pparg*)	Forward	5’-CCCTGGCAAAGCATTTGTAT-3’
	Reverse	5’-GAAACTGGCACCCTTGAAAA-3’
C/EBPα (*cebp*a)	Forward	5’-GCCAAGAAGTCGGTGGATAA-3’
	Reverse	5’- CCTTGACCAAGGAGCTCTCA-3’
HMGCR (*hmgcr*)	Forward	5′-CATGCTGCCAACATCGTCA-3′
	Reverse	5′-TTGTGGGACTTGCTTCCG-3′
ACC (*acaca*)	Forward	5′-ACAACGCAGGCATCAGAAGA-3′
	Reverse	5′-GCTGTGCTGCAGGAAGATTG-3′
ATGL (*pnpla2)*	Forward	5′- CTCGAGTTTCGGATGGAGAG-3′
	Reverse	5′- TGAGAATGGGGACACTGTGA-3′
HSL (*lipa*)	Forward	5′- TAGCTGGAGGTGGTTCTGCT-3′
	Reverse	5′-CCAGTTACCATCTTCAGTGTAG-3′

Note: Nrf-2, Nuclear factor erythroid 2-related factor 2; SOD, superoxide dismutase; SREBP-1, sterol regulatory element-binding protein 1; FAS, fatty acid synthase; PPAR--γ2, Peroxisome proliferator-activated receptor gamma 2; C/EBPα, CCAAT (cytosine-cytosine-adenosine-adenosine-thymidine)-enhancer-binding proteins; HMGCR, 3-hydroxy-3-methyl-glutaryl-coenzyme A reductase; ACC, Acetyl CoA Carboxylase; ATGL, Adipose triglyceride lipase; HSL, Hormone-sensitive lipase.

### Histopathological assessment of liver and pancreas sections

The liver and pancreas tissues were fixed in neutral buffered formalin (NBF) for several days before being placed in a paraffin block. These tissues were then treated with graded xylene and embedded in the paraffin. Paraffin-embedded tissues were sliced at 5 micro-meter thickness with a rotary microtome to analyze the microscopic characteristics of hepatic tissues. All these sections had been stained with hematoxylin and eosin (H and E). H and E staining revealed the congestion of the bile ducts, blood vessels, vascular linings, inflammatory mononuclear cell infiltration around the vascular region, necrotized zone, and white fat droplet deposition in the liver section. The big white fat droplet deposition can be used for steatosis grading. Steatosis was also graded in every pair of animals following a previously described technique [44]. Steatosis scores were graded as follows: (5% =  0; 5-33% =  1; 33-66% =  2; > 66% =  3); lobular inflammation was graded as follows: (none =  0; 2 foci =  1; 2-4 foci =  2; > 4 foci =  3); and hepatocellular ballooning was graded as follows: (none =  0; few =  1; prominent =  2). In each section, there were five field areas were recorded and at least three rats from each group were used to randomly assess every attribute. Here, n =  3 rats were selected for histopathological imaging, which was calculated using PowerG software [41].

The existence and intensity of fibrosis in the liver tissue sections were examined using picrosirius red staining (n = 3 rats). Furthermore, a third set of staining was also done for collagen deposition, known as Trichrome Milligan’s stain (n = 3 rats). Using free Image-J software (downloaded from the NIH website, USA), the percentage of fibrosis was assessed [40]. Using a light microscope (Zeiss Axioscope, Carl Zeiss Meditec AG, Jena, Germany), the stained sections were observed and captured on camera at either a 10 or 40x magnification for subsequent analysis.

### Statistical analysis

All values were calculated as the mean ± SD (standard deviation). The means of all groups were compared using one-way ANOVA, and specific groups that differed significantly from the rest were found using Tukey’s multiple comparison test. Graph Pad Prism software (Version 9) was used to conduct all statistical analyses. A *p-value* less than 0.05 was regarded as statistically significant.

## Results

### HPLC analysis of ethanolic extract of purple potato

**Fig 1:**
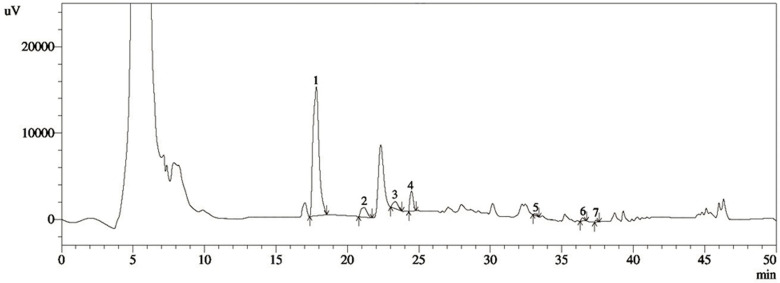
HPLC chromatogram for the ethanol extract of purple potato. The extract contains several polyphenolic compounds such as (1) 3,4-Dihydroxybenzoic acid (2) Catechin hydrate; (3) Catechol, (4) (-) Epicatechin, (5) trans-Ferulic acid, (6) Rosmarinic acid and (7) Myricetin. The phenolic compound found in the purple potato extract was calculated from the corresponding standard curve and was presented as the mean±SD as shown in Table 3.

HPLC was used to evaluate the presence of several phenolic components in the ethanolic extract of purple potato on both a qualitative and quantitative level. Phytochemicals present in the purple potato are separated based on their retention time in the applied solvent system, which is demonstrated in Fig 1. During a run time of 40 minutes, the HPLC-DAD system showed peaks of the phenolic compounds like 3,4-dihydroxybenzoic acid, (-) epicatechin, catechin hydrate, catechol, trans-ferulic acid, rosmarinic acid, and myricetin in the purple potato extract. For confirmation of the presence of a specific polyphenol, the HPLC injection was repeated three times, and one representative chromatogram out of those three repeated investigations of the extract is shown in Fig 1. The standard deviation values of three repetitions were used to measure each of the polyphenol compounds that was detected in the ethanolic extract of the purple potato (Table 3).

**Table 3:** Amount of different polyphenolic molecules in the ethanolic extract of purple potato.

Compounds	Purple Potato (mg/100 g dry extract)
Gallic acid	---
3,4-Dihydroxybenzoic acid	44.31 ± 0.21
Catechin hydrate	18.06 ± 0.46
Catechol	5.19 ± 0.06
(-) Epicatechin	15.48 ± 0.48
Caffeic acid	---
Vanillic acid	---
Syringic acid	---
Rutin hydrate	---
p-Coumaric acid	---
trans-Ferulic acid	0.50 ± 0.04
Rosmarinic acid	1.24 ± 0.11
Myricetin	0.37 ± 0.03
Quercetin	---
trans-Cinnamic acid	---
Kaempferol	---

### Effects of purple potato extract on bodyweight, water intake, calorie intake, liver wet weight, and lipid profile of HF diet-fed rats

**Fig 2:**
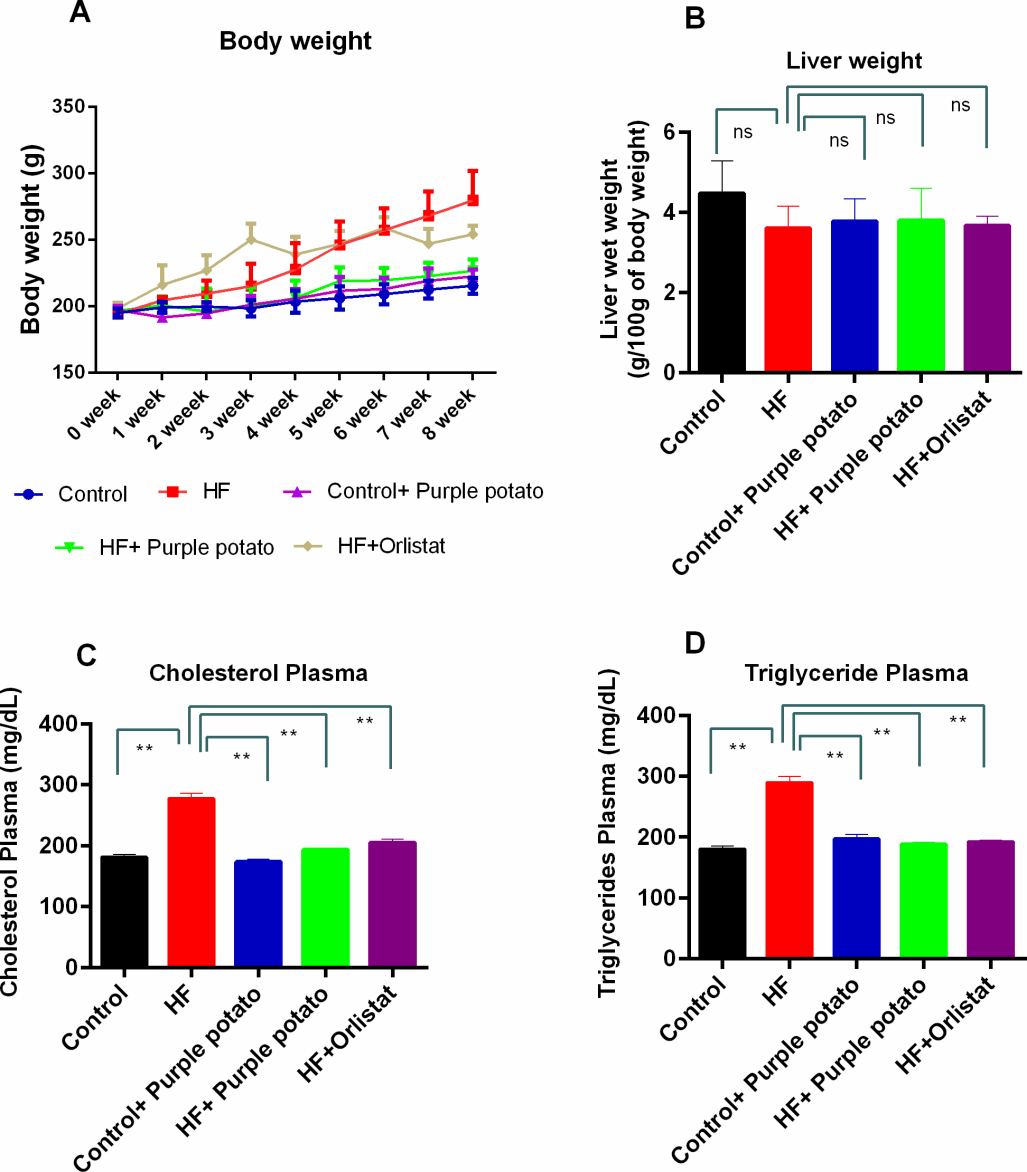
Effect of purple potato extract on body weight and lipid profile in HF diet-fed rats. (A) weekly body weight; (B) Liver wet weight; (C) Cholesterol in Plasma; and (D). Triglyceride in plasma. Data are presented as mean ±  SD, n =  6. For the statistical analysis, one-way ANOVA with Tukey’s multiple comparison test was performed. Statistical significance was considered as *p* ≤ 0.05 in all cases. Asterisks mark (**) represents *p* ≤  0.01.

Table 4 shows the beginning and eventual body weights of all rat groups. The ultimate body weights in the HF diet-fed rats were considerably higher (*p* ≤ 0.01) than the rats in the control group. Purple potato extract prevented HF-diet-induced body weight gain significantly (*p* ≤ 0.05) (Table 4). The weekly body weight gain of different groups of rats is presented in Fig 2A. It is observed that the HF diet increased the body weight compared to the control rats (Fig 2A). Purple potato extract administration for 8 weeks in HF diet-fed rats prevented the rise of body weights significantly (Fig 2A).

**Table 4:** Effect of purple potato on bodyweight and food and water consumption.

Parameters	Control	HF	Control + Purple potato	HF + Purple potato	HF + Orlistat
**Initial body weight (g)**	195.0 ± 3.27	194.2 ± 2.79	197.3 ± 2.92	196.8 ± 1.95	198.5 ± 4.43
**Final body weight (g)**	215.5 ± 6.02^*^	279.8 ± 22.27#	222.5 ± 5.47^*^	226.7 ± 8.60^*^	254.3 ± 6.26^*^
**Energy intake** **(Kelojouls/day)**	577.3 ± 56.54^*^	706.9 ± 51.50#	496.9 ± 57.66^*^	406.8 ± 74.55^*^	542.3 ± 106.39^*^
**Water intake mL/d**	20.14 ± 2.70	20.27 ± 1.97	15.42 ± 2.26	22.25 ± 3.91	22.09 ± 5.26

The data is provided with the mean ±  standard deviation (SD), n = 6 in each group. One-way ANOVA with Tukey’s multiple comparison test was the method employed for statistical analysis. The comparison was made Asterisk mark

*Versus hashtag mark # which represents significant difference at *p* < 0.01 level.

The calorie intake in HF diet-fed rats increased significantly (*p* ≤ 0.01) (706.9 ± 51.50 KJ/day) compared to the control rats (577.3 ± 56.54 KJ/day) (Table 4). The purple potato extract administration reduced the energy intake in HF diet-fed rats (406.8 ± 74.55 KJ/day) and control rats (496.9 ± 57.66 KJ/day) significantly (*p* ≤ 0.01) (Table 4). The orlistat treatment also decreased the energy intake (542.3 ± 106.39 KJ/day) significantly (*p* ≤ 0.01) compared to the HF diet-fed rats (Table 4).

The livers from each of the euthanized rats were collected at the end of the experiments and weighed by a precision balance. The wet weight of each liver was normalized to the body weight and presented as weight/100 g bodyweight. The liver weights of rats among the groups were not changed significantly (Fig 2B). Consistent with increased body weight, the HF diet substantially (*p* ≤ 0.01) elevated plasma levels of total cholesterol (TC) and triglycerides (TG) (Fig 2C and 2D) levels. Purple potato extract substantially (*p* ≤ 0.01) decreased this HF diet-induced elevated plasma TC and TG levels (Fig 2C and 2D). This decline of TC and TG was comparable to the reduction of those two harmful lipids by orlistat (Fig 2C and 2D).

### Effect of purple potato extract on the deposition of adipose tissues in HF diet-fed rats

**Fig 3:**
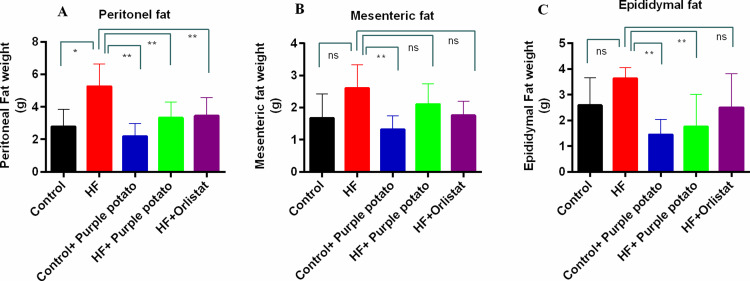
Effect of purple potato (*Solanum tuberosum L.)* on fat deposition in HF diet-fed rats. (A) Peritoneal, (B) mesenteric, and (C) epididymal fat deposits. Data are displayed as mean±SD, n = 6. Tukey’s multiple comparison test was used in conjunction with one-way ANOVA for statistical analysis. Statistical significance was considered as *p* ≤ 0.05 in all cases. Asterisks mark (**) represents *p* ≤  0.01, ns- not significant.

The peritoneal, mesenteric, and epididymal fat deposition in different groups of rats is presented in Fig 3A–3C, respectively. The peritoneal fat deposition was significantly increased (*p* ≤ 0.01) in the HF diet-fed rats (Fig 3A). Treatment with either purple potato or orlistat significantly (*p* ≤ 0.01) reduced HF diet-induced peritoneal fat accumulation (Fig 3A). Furthermore, as compared to the control group, rats in the control +  purple potato group also exhibited reduced fat accumulation in the peritoneal region, which is not significantly different (Fig 3A).

However, the HF diet did not increase the mesenteric fat deposition compared to the control rats (Fig 3B). Administration of purple potato extract in HF diet-fed rats did not lower the mesenteric fat weight (Fig 3B). Orlistat administration significantly lowered the mesenteric fat weight (*p* ≤ 0.01) in HF diet-fed rats.

Similarly, in this study, the HF diet also did not change the epididymal fat deposition compared to the control rats. The epididymal fat deposition was significantly (*p* ≤ 0.01) reduced by purple potato extract treatment (Fig 3C). Purple potato extract treatment also reduced the epididymal fat deposition in control rats (Fig 3C). However, orlistat administration did not lower the epididymal fat weight significantly in HF diet-fed rats (Fig 3C).

### Effect of purple potato extract on glucose tolerance as presented by oral glucose tolerance test (OGTT) in HF diet-fed rats

**Fig 4:**
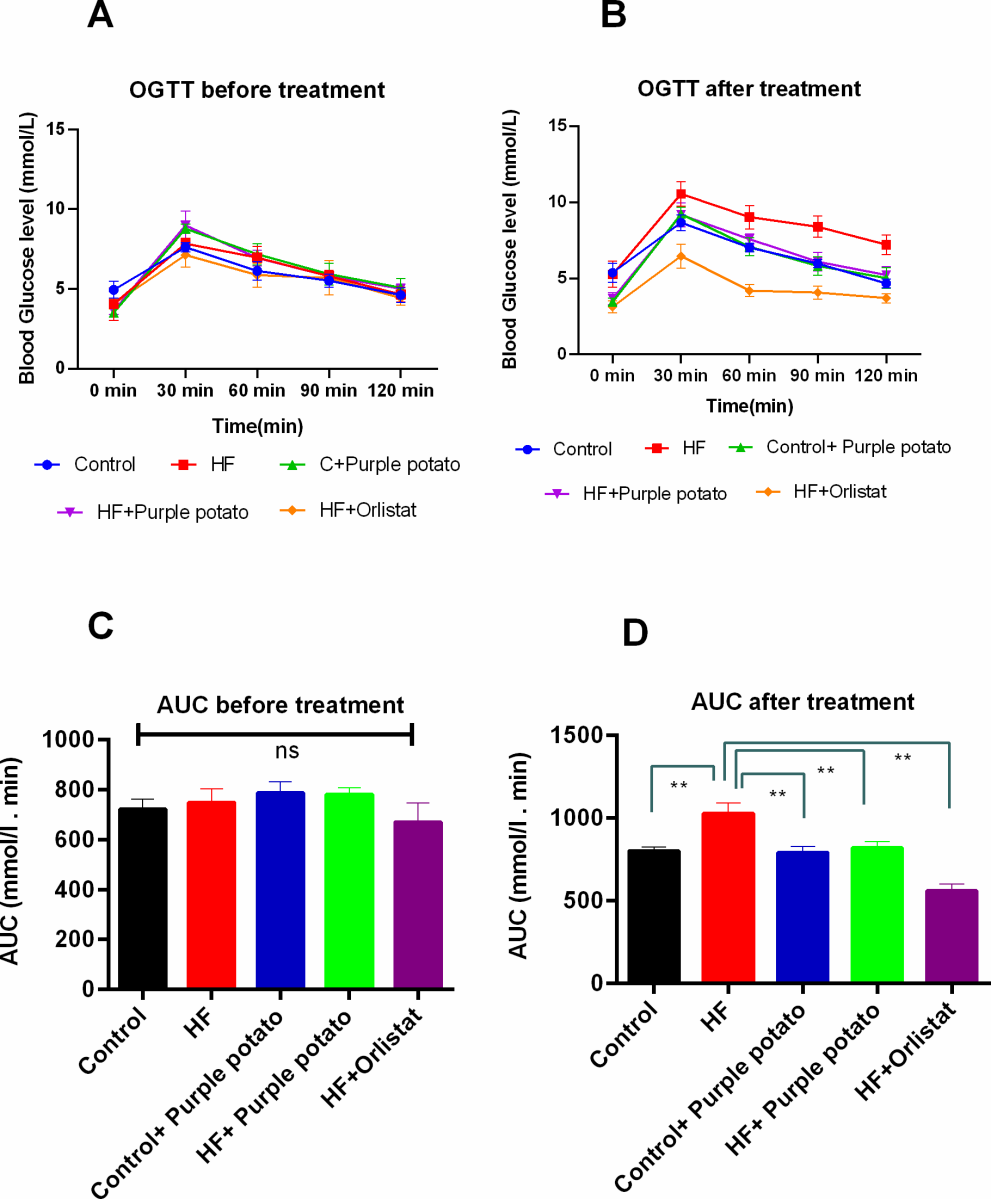
Oral Glucose Tolerance Test (OGTT) was conducted on the first day and the day before the rats were sacrificed. Here, the OGTT was conducted before (A) and after (B) the treatment. The area under the curve (AUC) is also represented in (C) before the treatment and (D) after the treatment. All values were presented using the mean ± SD, n =  6. One-way ANOVA was used for the statistical evaluation, and Tukey’s multiple comparisons test was used to compare each of the groups. Statistical significance was considered as *p* ≤ 0.05 in all cases. Asterisks mark (**) represents *p* ≤ 0.01.

People with obesity possess higher fasting blood glucose levels. An OGTT was done, before the commencement of the HF diet feeding and at the end of the HF diet feeding (after 56 days), to examine the ability to metabolize glucose by the experimental animals. Before the commencement of the feeding, the fasting plasma glucose concentration in all groups was around 4 mmol/L with no significant differences. In the post-treatment OGTT, all the groups showed similar levels of glucose except the HF diet-fed group (Fig 4B). This pattern of blood glucose indicates that the HF diet-induced hyperglycemic condition can be ameliorated by the extract of purple potato. The AUC of the OGTT test at the beginning of the study showed no significant differences among the groups studied (Fig 4C). However, the AUC data found at the end of the study showed significant differences (*p* ≤ 0.01) among the groups (Fig 4D). HF diet-fed rats showed significantly higher AUC compared to the control rats (Fig 4D). Purple potato extract and orlistat treatment in HF diet-fed rats significantly lowered the AUC compared to the HF diet-fed rats alone (Fig 4D). Purple potato extract treatment did not alter the AUC in control rats (Fig 4D).

### Effect of purple potato extract on the pancreatic morphology and insulin level in the plasma of HF diet-fed rats

**Fig 5:**
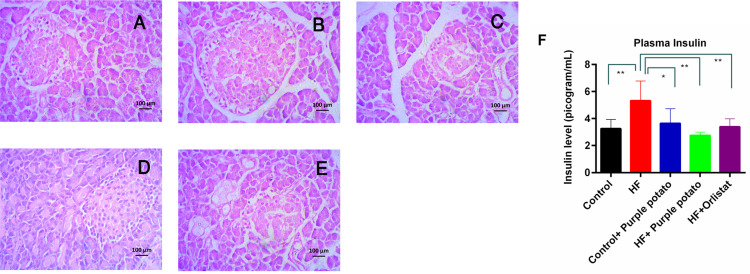
Effect of purple potato (*Solanum tuberosum* L.) on pancreatic islets and insulin level in high-fat diet fed rats. (A) control; (B) HF; (C) control+purple potato; (D) HF+purple potato, (E) HF+orlistat; (F) Plasma insulin level. Data are presented as mean ± SD, n =  6. Statistical analysis was done by one-way ANOVA and comparisons among the groups were done following Tukey’s multiple comparisons test. Statistical significance was considered as *p* ≤ 0.05 in all cases. Asterisks mark (**) represents *p* ≤  0.01.

The pancreatic islets were studied histologically to determine the preventive effects of purple potato extract on HF diet-fed rats. The pancreatic tissues were collected at the end of the study after sacrifice. The tissues were then processed and stained with hematoxylin and eosin. The pancreatic islets of the control group were seen as normal (Fig 5A), but the pancreatic islets of the HF group revealed hypertrophic alterations which were correlated with increased insulin levels in the plasma (Fig 5B). The HF diet-fed rats treated with either purple potato extract or with orlistat demonstrated no alterations in pancreatic morphology and in the plasma insulin levels compared to HF diet-fed rats (Fig 5C and 5D).

### Effect of purple potato extract on the hepatic enzyme’s activities in plasma of HF diet-fed rats

**Fig 6:**
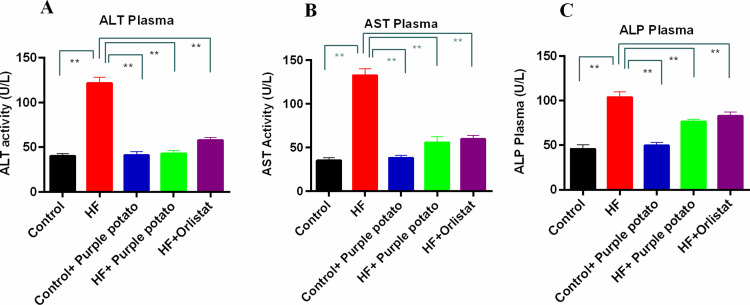
Effect of purple potato (Solanum tuberosum L.) on hepatic marker enzymes. (A) ALT Plasma. (B) AST Plasma and (C) ALP in plasma. Mean ± SD was used to express all values, where n = 6. Statistical analysis was done by one-way ANOVA and comparisons among the groups were done following Tukey’s multiple comparisons test. Statistical significance was considered as *p* ≤ 0.05 in all cases. Asterisks mark (**) represents *p* ≤  0.01.

Due to the consumption of an HF diet, fat deposition in the hepatic tissue progresses quickly, which triggers hepatic injury. The activity of liver enzymes such as ALT, AST, and ALP was measured in order to evaluate hepatic damage. Fig 6 depicts changes in hepatic enzyme activities. The ALT, AST, and ALP activities were significantly (*p* < 0.01) higher in rats consuming HF diets in comparison to the control group (Fig 6A–6C). Purple potato extract treatment in HF-diet-fed rats prevented the increased ALT, AST, and ALP activities (Fig 6A–6C) in plasma. Similarly, the treatment of orlistat in HF-diet-fed rats also prevented the elevation of these three enzymes (Fig 6A–6C). Moreover, treatment with purple potato extract and orlistat had no unwanted impact on the liver enzyme activities in the control rats (Fig 6A–6C).

### Effect of purple potato extract on oxidative stress markers in plasm and liver of HF diet-fed rats

**Fig 7:**
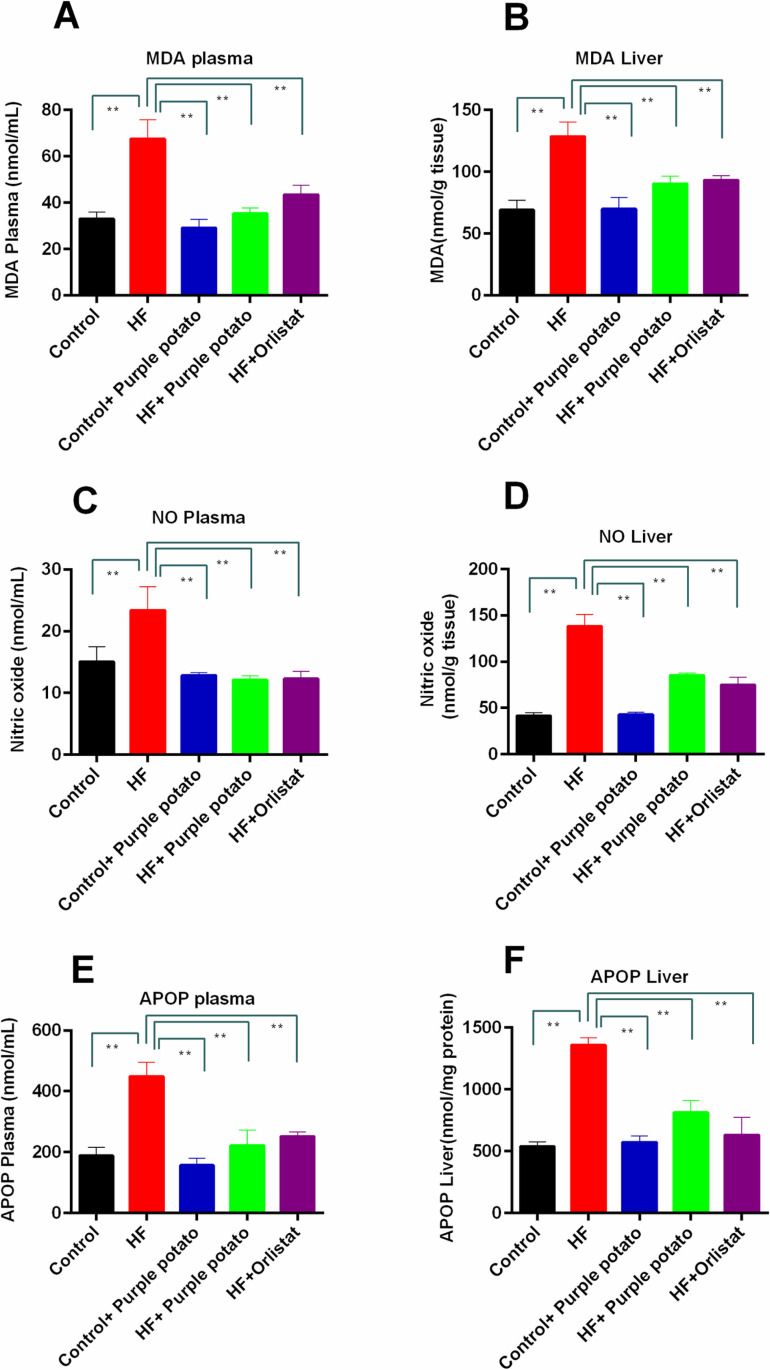
Effect of purple potato (*Solanum tuberosum L.)* on oxidative stress markers in HF diet-fed rats. (A) MDA in plasma. (B) MDA in the liver. (C) NO in plasma. (D) NO in the liver. (E) AOPP in plasma. (F) AOPP in the liver. Mean ±  SD was used to express all values, where n =  6. Statistical analysis was done by one-way ANOVA and comparisons among the groups were done following Tukey’s multiple comparisons test. Statistical significance was considered as *p* ≤ 0.05 in all cases. Asterisks mark (**) represents *p* ≤  0.01.

The levels of MDA, NO, and AOPP were used in assessing the level of oxidative stress. Fig 7 depicts the findings from each of the groups. MDA concentrations in the serum and the hepatic tissue of HF diet-fed rats were substantially (*p* ≤ 0.01) greater than those in the control group (Fig 7A and 7B). The amounts of MDA in the plasma and the liver of rats in the HF +  purple potato group were significantly reduced (*p* ≤ 0.01) than the HF diet-fed rats. This MDA lowering capability of purple potato extract was almost equal to the orlistat treatment (Fig 7A and 7B). Similarly, NO and AOPP levels were also found to be significantly (p ≤ 0.01) higher in plasma and in the hepatic tissues of HF diet-fed rats than in the control rats. This HF-diet-mediated increased levels of NO and AOPP were suppressed significantly (*p* ≤ 0.01) due to the feeding of purple potato extract (Fig 7C–7F). Orlistat and purple potato extract extracts were found to be similarly effective in all cases in lowering the HF-diet-induced increased oxidative stress. However, feeding purple potato extract to the control group did not affect the level of MDA, NO, or AOPP levels.

### Effect of purple potato extract on the antioxidant enzymes activities in plasma and liver of HF diet-fed rats

**Fig 8:**
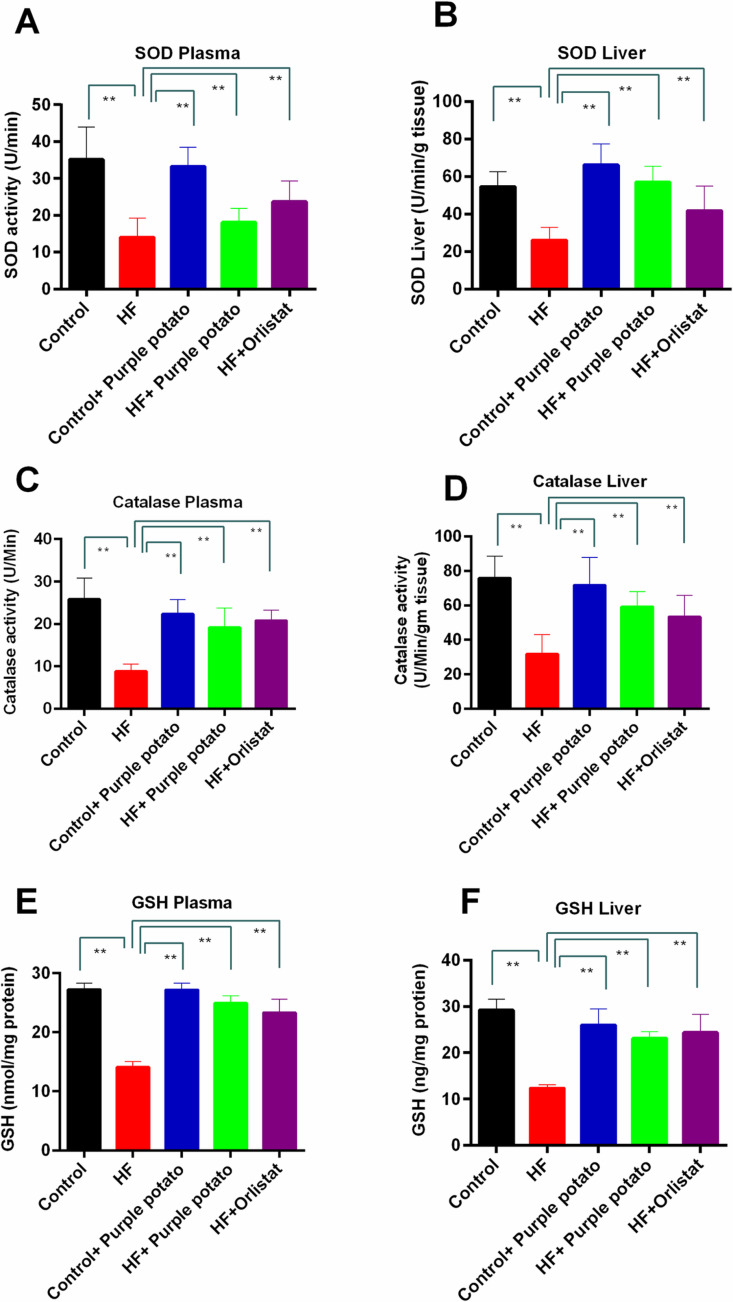
Effect of purple potato (*Solanum tuberosum L.)* on antioxidant enzymes in HF diet-fed rats. In the plasma and liver of high-fat diet-induced obese rats, the antioxidant enzymes superoxide dismutase (SOD) and catalase, as well as the antioxidant glutathione (GSH) levels, were assessed. (A) SOD plasma; (B) SOD liver, (C) Catalase plasma; (D) catalase liver; (E) GSH plasma and (F) GSH liver. The values are shown as mean±SD, n = 6. Statistical analysis was done by one-way ANOVA and comparisons among the groups were done following Tukey’s multiple comparisons test. Statistical significance was considered as *p* ≤ 0.05 in all cases. Asterisks mark (**) represents *p* ≤  0.01.

The enzymatic defense against oxidative stress involves a coordinated action of various antioxidant enzymes and GSH. SOD catalyzes the dismutation of the superoxide radical (O_2_^-^) into oxygen (O_2_) and hydrogen peroxide (H_2_O_2_), which is one of the first enzymes that play a crucial role in reducing oxidative stress. This section of our study was aimed at exploring the activity of antioxidant enzymes such as SOD and catalase and the level of GSH in the plasma and liver homogenates. The activity of SOD and catalase was found significantly (*p* ≤ 0.01) low in plasma and liver in the HF group than in the control group (Fig 8A and 8B). This decline in SOD activity was restored by purple potato extract administration in both plasma and liver samples (Fig 8A and 8B). In HF +  orlistat-fed animals, orlistat treatment also increased SOD and catalase activity comparably (Fig 8). However, feeding purple potato to the control group caused no significant change in the plasma and liver SOD and catalase activity (Fig 8B and 8A). In agreement with these findings, HF diet-fed rats showed reduced GSH levels in the plasma (*p* ≤ 0.01) and liver tissue (*p* ≤ 0.01) in comparison to the control rats (Fig 8E and 8F). Purple potato extract feeding also restored the GSH level in the plasma and liver of HF diet-fed rats (Fig 8E and 8F).

### Effect of purple potato extract on the gene expression of antioxidant enzymes in liver HF diet-fed rats

**Fig 9:**
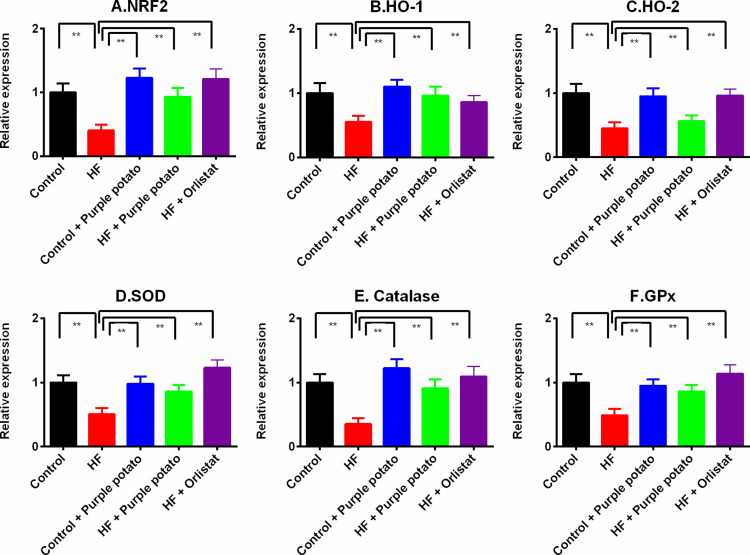
Effect of purple potato (*Solanum tuberosum L.* on antioxidant enzyme activity genes expression in the liver of HF diet-fed rats. Here, (A) Nuclear factor erythroid 2-related factor 2 (NRF 2); (B) Heme oxygenase-1 (HO-1). (C) Heme oxygenase-1 (HO-2). (D) Superoxide dismutase (SOD); (E) Catalase; (F) Glutathione peroxidase (GPx). The values are shown as mean±SD, n = 6. Statistical analysis was done by one-way ANOVA and comparisons among the groups were done following Tukey’s multiple comparisons test. Statistical significance was considered as *p* ≤ 0.05 in all cases. Asterisks mark (**) represents *p* ≤  0.01.

The findings of the present investigation on antioxidant enzyme activities in plasma and liver tissue indicate that these enzymatic functions are lowered in HF diet-fed animals. By considering these feeble enzyme activities, we additionally investigated the gene expression pattern of a few factors and enzymes related to antioxidant defense which are: nuclear factor erythroid 2-related factor (Nrf-2), heme oxygenase-1 (HO-1), heme oxygenase-2 (HO-2), SOD, catalase, and GPx. Fig 9 depicts the expression of these antioxidant genes. This investigation finds that the relative expression of the genes Nrf-2, HO-1, and HO-2 are significantly lowered in the liver of HF diet-fed rats compared to the control rats (Fig 9A–9C). Purple potato extract and orlistat treatment restored the mRNA levels of Nrf-2, HO-1, and HO-2 in the liver of HF diet-fed rats (Fig 9A–9C). In line with this investigation, SOD, catalase, and GPx mRNA expression were also analyzed, and reveled that these mRNA expressions were lowered in the liver of HF diet-fed rats substantially (*p ≤ 0.01*) than in the control groups. In comparison to the HF diet-fed group, rats in the HF +  purple potato group showed a greater relative expression of SOD, catalase, and GPx mRNA expressions (*p* < 0.01). The HF +  Orlistat group also showed augmented gene expression of these three vital enzymes in the liver (Fig 9D–9F).

### Effect of purple potato extract on the gene expression of fat metabolism-related factors and enzymes in the liver of HF diet-fed rats

**Fig 10:**
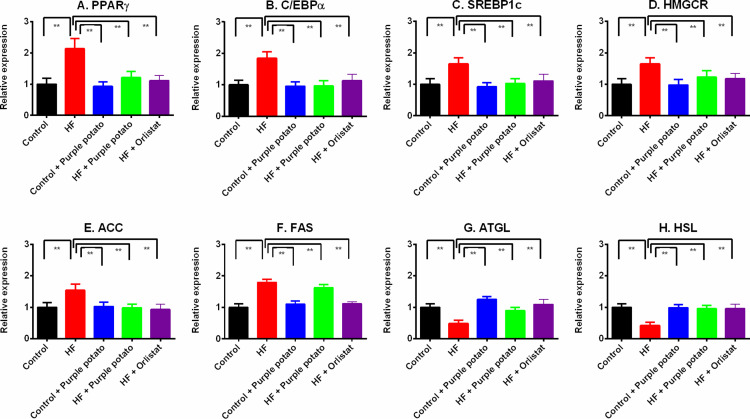
Effect of purple potato (*Solanum tuberosum L.)* on fat metabolizing gene expression in the liver of high-fat diet-induced obese rats. (A) Peroxisome proliferator-activated receptor gamma (PPARγ); (B) CCAAT enhancer binding protein alpha (C/EBPα); (C) Sterol regulatory element binding protein 1c (SREBP1c); (D) Anti-3-hydroxy-3-methylglutaryl-CoA reductase (HMGCR) (E) Acetyl-CoA carboxylase (ACC); (F) Fatty acid synthase *(FAS);* (G) Adipose triglyceride lipase (ATGL); (H) Hormone-sensitive lipase (HSL). The values are shown as mean±SD, n = 6. Statistical analysis was done by one-way ANOVA and comparisons among the groups were done following Tukey’s multiple comparisons test. Statistical significance was considered as *p* ≤ 0.05 in all cases. Asterisks mark (**) represents *p* ≤  0.01.

To comprehend how treatment with purple potato extract affected lipid metabolism in the liver, we investigated the expression of factors and enzymes associated with lipogenesis and fat metabolism. The HF diet-fed rats displayed significantly (*p* ≤ 0.01) higher mRNA levels of pro-adipogenic factors such as peroxisome proliferator-activated receptor gamma (PPARγ), CCAAT enhancer binding protein alpha (C/EBPα), sterol regulatory element binding protein 1c (SREBP1c), and HMG CoA reductase (HMGCR) than the control group. Purple potato extract feeding caused significant normalization of these mRNA transcripts levels in the liver of HF diet-fed rats (Fig 10A–10D). Orlistat treatment in HF diet-fed rats also normalized the PPARγ, C/EBPα, SREBP1c, and HMGCR mRNA expression in the liver. However, rats in the control +  purple potato group showed no significant changes in the expression of the above-mentioned mRNA transcripts in comparison to the control group (Fig 10A–10D).

Additionally, we also explored the mRNA expression of fat metabolizing enzymes such as acetyl-CoA carboxylase (ACC), fatty acid synthase *(FAS),* adipose triglyceride lipase (ATGL), and hormone-sensitive lipase (HSL) in the liver of all groups of rats. HF diet-fed rats showed higher levels of ACC and FAS mRNA expression compared to the control (*p* ≤ 0.01) (Fig 10), however, purple potato extract and orlistat suppressed the ACC and FAS mRNA expression in the liver of HF diet-fed rats (Fig 10G, 10H). The ATGL and HSL mRNA expression were found to decline significantly (*p* ≤ 0.01) in the liver of HF diet-fed rats, which were further restored by purple potato extract administration (Fig 10E and 10F). Orlistat treatment also restored the ATGL and HSL mRNA expression in the liver of HF diet-fed rats (Fig 10G and 10H).

### Effect of purple potato extract on histology of liver of HF diet-fed rats

**Fig 11:**
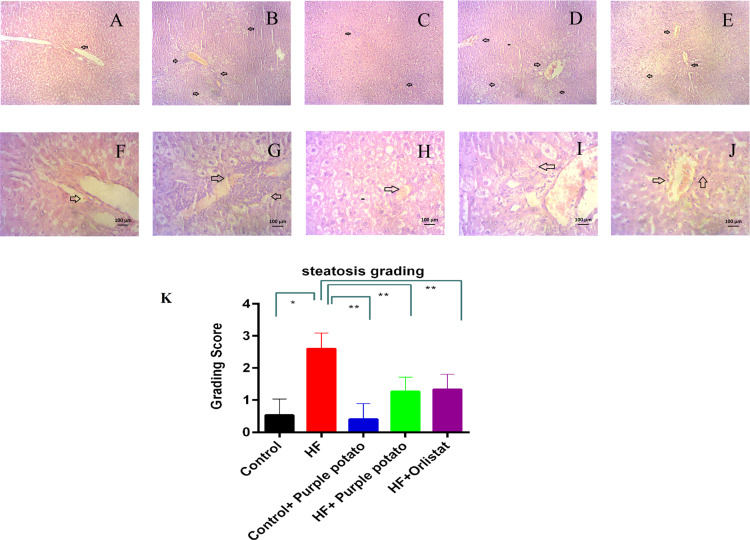
Effect of purple potato extract (*Solanum tuberosum L*.) on hepatic inflammatory cells infiltration and fat deposition in HF diet-fed rats. Hematoxylin and Eosin staining was performed on liver tissue sections. (A, F) control liver segment, the hepatocytes were encompassed by the bile ducts in a normal form; (B, G) in the HF-high magnification liver section, fat droplet deposition and inflammation appeared readily apparent; (C, H) Control + purple potato, which displayed normal architecture in the liver section; (D, I) HF + purple potato, which demonstrated reduced or lacking fat droplet deposition and inflammation around the bile ducts; and (E, J) HF+Orlistat, which displayed reduced inflammatory cells and a reduced fat deposition. The upper panel represents 10x magnification and the lower panel represents 40X magnification. K represents the steatosis grading score of different groups of rats. Data are presented as mean±SD, where n = 3. For statistical analysis, One-way ANOVA was performed, and the Tukey test was done as a post hoc test to compare the means of every group present in this study. Statistical significance was considered as *p* ≤ 0.05 in all cases. Asterisks (**) mark represents *p* ≤ 0.01.

Hepatic steatosis and necrosis due to HF diet feeding in rats are presented in Fig 11. Animals in the control group did not exhibit steatosis or fat droplets in the liver (Fig 11A) and showed normal structural orientation. In contrast, HF diet-fed rats showed increased fat droplet deposition and steatosis (Fig 11B). The scoring system revealed that the steatosis score is significantly (*p* ≤ 0.01) higher in the HF diet-fed rats compared to the control rats (Fig 11K). However, HF +  purple potato rats displayed reduced steatosis and fat droplet accumulation (Fig 11D). Additionally, HF +  purple potato rats showed a lower steatosis score than HF diet-fed rats (Fig 11K). HF +  orlistat group also showed a decreased steatosis in comparison to HF diet-fed rats (Fig 11K). In the control +  purple potato group liver histology showed normal architecture of hepatic tissue (Fig 11C).

**Fig 12:**
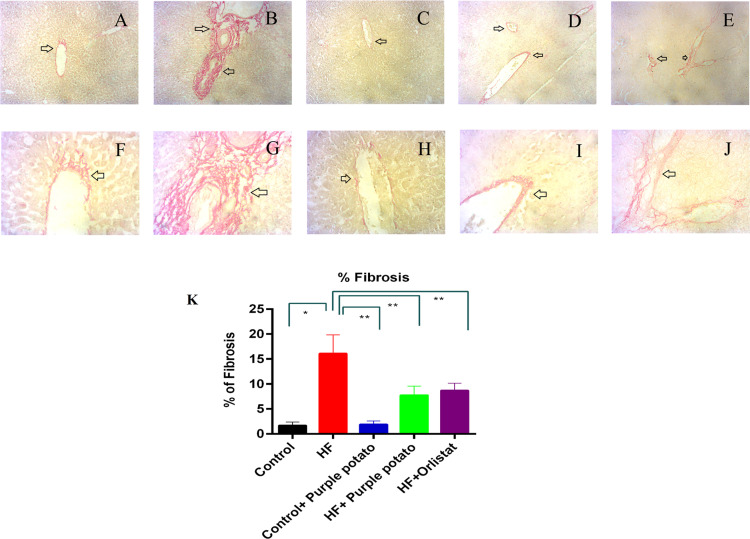
Effect of purple potato extract (*Solanum tuberosum L*.) on hepatic fibrosis in HF diet-fed rats. Sirius red staining was used on liver tissue sections. (A, F) control liver segment showed normal collagen deposition around the bile ducts and blood vessels; (B, G) HF, collagen deposition around the bile ducts and blood vessels are distinctive; (C, H) Control + purple potato, which displayed normal architecture in the liver section as in control; (D, I) HF + purple potato, which demonstrated reduced collagen deposition around the bile ducts and blood vessels; and (E) HF+Orlistat, which displayed less collagen deposition around the bile ducts and blood vessels. The upper panel represents 10x magnification and the lower panel represents 40X magnification. K represents the % fibrosis of different groups of rats. Data are presented as mean±SD, where n = 3. For statistical analysis, One-way ANOVA was performed, and the Tukey test was done as a post hoc test to compare the means of every group present in this study. Statistical significance was considered as *p* ≤ 0.05 in all cases. Asterisks (**) mark represents *p* ≤ 0.01.

Fig 12 shows the liver section after being stained with Sirius red for collagen deposition. The collagen deposition in the HF diet-fed rats was significantly increased in comparison to the control rats (Fig 12B). Purple potato extract treatment in HF diet fed rats prevented the collagen deposition and fibrosis in the liver (Fig 12D). Additionally, the HF +  orlistat group also showed a decreased collagen deposition in comparison to HF diet-fed rats (Fig 12E). Normal collagen distribution was found in control +  purple potato rats (Fig 12C).

**Fig 13:**
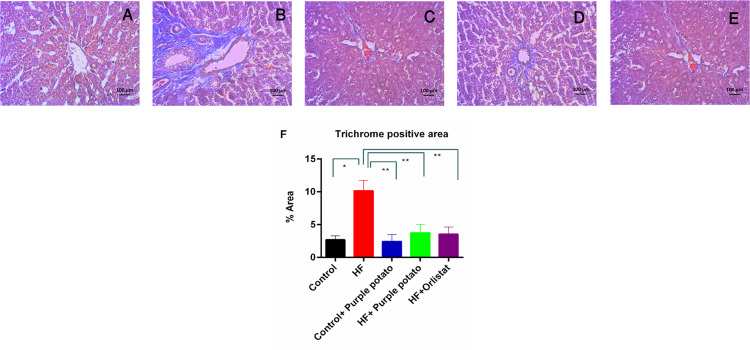
Effect of purple potato extract (*Solanum tuberosum L*.) on hepatic Trichrome positive area in HF diet-fed rats. Trichrome Milligan’s staining was used on liver tissue sections. (A) control liver segment, the bile ducts were surrounded by hepatocytes in a standard pattern; (B) in the HF, increased Trichrome positive area were seen around the bile ducts; (C) Control + purple potato, which displayed consistent structure in the liver section as in control; (D) HF + purple potato, which displayed decreased or absent of Trichrome positive area around the bile ducts; and (E) HF+Orlistat, which displayed decreased or absent of Trichrome positive area around the bile ducts. 40X magnification. F, represents the Trichrome positive % area of different groups of rats. Data are presented as mean±SD, where n = 3. For statistical analysis, the One-way ANOVA was performed, and the Tukey test was done as a post hoc test to compare the means of every group present in this study. Statistical significance was considered as *p* ≤ 0.05 in all cases. Asterisks (**) mark represents *p* ≤ 0.01.

Furthermore, Trichrome Milligan’s staining on the liver section also confirmed the collagen deposition and fibrosis in HF diet-fed rats compared to the control rats (Fig 13 B and Fig 13F). Purple potato extract administration prevented fibrosis and lowered Trichrome positive area in the liver of HF diet-fed rats (Fig 13D and Fig 13F). A similar result was also found with the treatment of orlistat in HF diet-fed rats and showed reduced collagen deposition (Fig 13E). Normal collagen distribution and Trichrome positive area were found in control +  purple potato rats (Fig 13C and 13F).

## Discussion

Obesity generates a variety of diseases, including hyperlipidemia, NAFLD, hypertension, and cardiovascular dysfunction [45]. HF diet is used to develop animal models for obesity, exhibits consequences comparable to those found in people, are often researched [46]. In this investigation, an obese rat model was developed using an HF diet that culminated in the establishment of oxidative stress and inflammation followed by dyslipidemia, NAFLD, and related complications. This investigation also showed that purple potato extract treatment ameliorated oxidative stress, dyslipidemia, and NAFLD in HF diet-fed rats.

An HF diet has been shown to significantly increase body weight because of its high energy content. Purple potato extracts inhibited body weight gain in rats fed an HF diet. The study additionally showed that feeding rats with an HF diet increased the amounts of peritoneal fat, which is the major fat tissue in the animal body. Peritoneal fat is found in the abdomen, and prior studies have revealed that higher levels of peritoneal fat may be linked to the development of metabolic syndrome [47]. Mesenteric fat is found in the abdominal area around the intestines, and it has been proposed that both peritoneal and mesenteric fat can contribute to the development of fatty liver and insulin resistance by increasing the load of fatty acids in the liver [48]. According to the findings of this study, purple potato extract could be a possible dietary intervention for avoiding fat deposition and inhibiting fatty liver development. This investigation showed that purple potato extract treatment lowered peritoneal fat accumulation and may assist to prevent the development of fatty liver which are consistent with the previous report [49].

HF diet might make it more difficult for the body to utilize glucose for energy, leading to higher blood sugar levels. A prior study discovered that HF diet-fed rats showed higher blood glucose levels [50]. This is most likely because saturated fats in HF diets might hinder the body’s capacity to create and utilize insulin, a hormone that helps regulate blood sugar levels [51]. The present investigation demonstrated that rats fed an HF diet acquired glucose intolerance or hyperglycemia, which is consistent with prior research findings that link glucose intolerance and insulin resistance to obesity [52]. This observation was corroborated by data from the OGTT. At the start of the investigation, all groups of rats demonstrated normal glucose clearance at 120 minutes following the administration of glucose in fasting settings. The glucose metabolism was impacted following 8 weeks of HF diet feeding, with blood glucose levels persisting high at 120 minutes after the glucose dose. The concentrations of insulin were also observed to be increased in HF diet-fed rats with respect to the remaining groups tested in the present investigation. The hypertrophy of islets was also found in the pancreas in HF diet-fed rats. Purple potato extract prevented the hypertrophy of islets as well as normalized glucose utilization and insulin level, suggesting the ameliorating effect in glucose intolerance and insulin resistance.

Insulin resistance and glucose utilization may be a result of increased oxidative stress due to HF diet. ROS are molecules that can harm cells and are responsible for the development of oxidative stress. ROS can be created by the body as part of normal metabolism, but they can also be produced excessively under specific situations, such as a HF diet [52,53]. Insulin resistance, a condition in which the body’s cells become less receptive to the hormone insulin, can be caused by ROS [54]. Insulin is in the midst of assisting cells in absorbing glucose from the blood. When cells become insulin-resistant, glucose accumulates in the blood, which may lead to type 2 diabetes [55]. ROS can damage insulin receptors in adipose and skeletal muscle tissues, which are cellular structures that absorb glucose from circulation [56]. Purple potato extract has antioxidant capabilities, which means it can help safeguard cells from ROS damage. Ethanol extract of purple potato extract was found to possess a number of antioxidants such as 3,4-dihydroxybenzoic acid and other antioxidant compounds, which may be responsible for improved glucose tolerance and prevent hyperglycemia in rats fed a HF diet.

An earlier report suggests that rats fed an HF diet had significantly greater levels of oxidative stress indicators in plasma and liver, including MDA and NO [57]. Free radicals are unstable chemicals that can cause tissue and cell damage. Multiple processes may be involved in the higher levels of oxidative stress in HF-fed rats. The HF diet also boosted the activity of inducible nitric oxide synthase (iNOS) in the liver. The iNOS is an enzyme that generates NO, a biomolecule with numerous functions, including blood pressure regulation and inflammation [58]. When NO is created in excess, it can react with free radicals to form peroxynitrite, a highly reactive chemical that can cause cell damage [59]. The study found that purple potato extract significantly reduced all of the oxidative stress markers such as MDA and NO. Moreover, HF diet-fed rats showed declined antioxidant enzyme activities such as SOD and catalase. This finding is in line with the previous results showed that increased oxidative stress parameters are evident in HF diet-fed rats [49]. Purple potato extract restored the antioxidant enzyme activities in HF diet-fed rats. This result is supported by previous results showing that purple sweet potato (*Ipomoea batatas* L.) prevented oxidative stress and restored antioxidant enzymes in HF diet-fed rats [60].

We also investigated the expression of antioxidant genes such as *nrf2l2*, *sod1, cat*, and *gpx*. The results revealed that the HF diet-fed rats showed a considerably lower level of relative expression of antioxidant genes in the liver than the control rats. This implies that an HF diet can reduce the expression of antioxidant genes, contributing to oxidative stress. Purple potato extract was found to raise the relative expression of antioxidant genes in rats fed an HF diet; denoting that by activating antioxidant genes, purple potato extract may assist in protecting against oxidative stress. This study also revealed that purple potato extract may boost the expression of the *nrf212* gene, a key regulator of genes associated with oxidative stress [61]. The Nrf-2 is a transcription factor that regulates gene expression in antioxidant defense, detoxification, and cell survival [61]. The study’s findings are consistent with prior research that has demonstrated that an HF diet can lead to decreased *nrf2l2* gene expression and that antioxidant supplementation can boost *nrf2l2* gene expression [62].

An HF diet feeding in rats also raised plasma lipids, triglycerides, and cholesterol levels. This is because saturated fats in HF diets might raise cholesterol and triglyceride synthesis in the liver. The current study also shows that purple potato extract lowers triglyceride and cholesterol levels in rats fed an HF diet. This lipid profile-lowering effect of purple potato is attributed to the function of fat metabolizing gene expression in the liver. Previous research found that the expression of genes related to lipid metabolism and lipogenesis, such as *pparg* and *fasn*, is altered in the hepatic tissue of obese individuals [63]. The *pparg* and *fasn* genes are involved in the production of fatty acids [59]. Purple potato extract treatment lowered the expression of *pparg* and *fasn* genes in the liver, which could be responsible for the reduced triglyceride and cholesterol levels as well as decreased steatosis development in the liver. Further, other related genes such as *cebpa, srebf1, acaca,* and *hmgcr* levels were also increased in the liver of HF diet-fed rats, which was further normalized by purple potato extract treatment. This finding is also supported by the previous report showing that the Korean red ginseng formula decreased the expression of lipogenesis factors, namely *cebpa, srebf1* and *acaca* in rats [64].

ATGL and HSL are a pair of lipolytic enzymes that are associated with fighting the development of liver steatosis. HF diet-fed rats showed decreased *pnpla2* and *lipa* gene expression in the liver which could be attributed to the fat droplet deposition in hepatocytes. Earlier studies have proposed that hepatic overexpression of *pnpla2* and *lipa* may promote the oxidization of fatty acids and alleviate hepatic steatosis [65]. Purple potato and orlistat treatment in HF diet-fed rats showed increased relative mRNA expression of *pnpla2* and *lipa* genes in the liver. Moreover, histological staining revealed the reduced fat droplet and steatosis score in the liver of HF diet-fed rats.

HF diet leading to oxidative stress causes hepatic cell damage, necrosis, inflammatory cell infiltration, and fibrosis in the liver. Hepatic stellate cells are the main sources of the extracellular matrix protein mainly collagen release and may develop fibrosis [66]. This investigation revealed that an increased level of fibrosis was observed in the liver section of HF diet-fed rats. Antioxidants included in many dietary supplements may help to reduce inflammatory cell infiltration and fibrosis in the liver [49]. Purple potato extract treatment prevented fibrosis in the liver of HF diet-fed rats.

This study found that purple potato extract therapy helped reduce complications in HF diet-fed rats. Purple potato extract administration in control rats in this study indicated that there was no adverse reaction in terms of hepatic transaminase enzyme activities, which was not affected when compared to control rats. Furthermore, purple potato extract resulted in decreased body weight in control rats, which was related to declined fat accumulation in control rats. Previous investigations additionally delved into the hazardous effects of purple potato extract. According to a previous study, C57BL/6N mice receiving an ethanolic extract of purple potato for five weeks did not significantly increase the risk of developing cancer [67].

The result of this study shows great promise in preventing diet-induced obesity and related complications in the liver of rats. However, some limitations are also present in this study. First, the purple potato extract may contain other bioactive materials not identified in this study. Secondly, the male rats were used in this study which explains the stress and complications in male individuals only. The estrogenic action in females may have a preventive role in the development of inflammation and related pathophysiology [68]. Thus, female rats were excluded in this study. Thirdly, the Homeostatic Model Assessment for Insulin Resistance (HOMA-IR) was not performed in this study, which helps diagnose insulin resistance in diabetes conditions. However, the OGTT test in this study revealed glucose intolerance despite of high level of insulin presence in the plasma of HF diet-fed rats.

In summary, the current investigation showed that, in HF diet-fed rats, the purple potato extract inhibits obesity improves glucose intolerance and oxidative stress and modulates fat metabolizing gene expression. Thus, purple potato extract may be employed as an alternative therapeutic approach against obesity and metabolic syndrome. Toxicological investigations and clinical trials on humans should be warranted in future investigations.

## Supporting information

S1 File Methods of gas chromatographic analysis of fatty acids in control and high fat diet. (DOCX)

S1 Fig Gas chromatography chromatogram for fatty acids present in the control diet. (JPG)

S2 Fig Gas chromatography chromatogram for fatty acids in the high-fat diet. (JPG)

S1 Table Gas chromatography analysis of different fatty acids present in the control diet. (DOCX)

S2 Table Gas chromatography analysis of different fatty acids present in the HF diet. (DOCX)

S1 Data Biochemical analysis of purple potato project. (XLSX)

S2 Data Body weight food intake water intake of Jamalo 08.02.2022. (XLSX)

S3 Data HF+PP-1 fat metabolizing genes. (XLSX)

S4 Data HF+PP-2 oxidative stress genes. (XLSX)

S5 Data OGTT test purple potato. (XLSX)

S6 Data Organ weight purple potato. (XLSX)
