# Supplementary material for: Purple potato extract modulates fat metabolizing genes expression, prevents oxidative stress, hepatic steatosis, and attenuates high-fat diet-induced obesity in male rats
Source: PLoS One. 2025 Apr 1;20(4):e0318162. doi: 10.1371/journal.pone.0318162 (PMC11960900; doi:10.1371/journal.pone.0318162)
Supplement: S1 File — (DOCX) [file pone.0318162.s001.docx]

**Supplementary File 1**

**Methods of Gas Chromatographic Analysis of Fatty Acids in Control and High Fat Diet**

Fatty acid methyl esters (FAME) were prepared according to ISO 12966-2:2017(E) with some modifications (ISO, 2017; Lisa et al. 2024). In brief, approximately 50 mg of feed sample was placed in a 10 mL screw-capped test tube, and 2 mL of isooctane was added and shaken. Then, 0.1 mL of a 2 M methanolic potassium hydroxide solution was added and shaken vigorously for 1 min. After 2 min of rest, 2 mL of saturated sodium chloride solution was added and shaken properly. After phase separation, the isooctane layer was passed through an anhydrous sodium sulfate column to collect the FAME in a 2 mL wide-necked glass vial for GC analysis. A Thermo Scientific Gas Chromatograph system (Trace 1300, USA) coupled with a flame-ionized detector, an autosampler, and a highly polar fused silica capillary column (Rt-2560, Restek, Bellefonte, PA, USA) (100 m × 0.25 mm id × 0.25 μm film thickness) was used for fatty acid methyl ester separation. Ultrapure nitrogen gas at a constant flow rate of 1 mL/min was used as a carrier gas for GC analysis. The initial oven temperature was 150°C with a 10 min holding time. The temperature was then increased to 200°C at a rate of 5°C/min and held at this temperature for 10 min. After that, the temperature increased at 10°C/min to 240°C and held for 20 min. The split injection mode was used for this analysis, and the split ratio was 20:1. Both injection temperature and detector temperature were 250°C. The separated peaks were identified by using Supelco 37 Component FAME MIX (Supelco, Bellefonte, PA, USA), a Linolenic acid methyl ester isomer mix, and a Linoleic acid methyl ester mix (Supelco, Bellefonte, PA, USA).

**Results:**


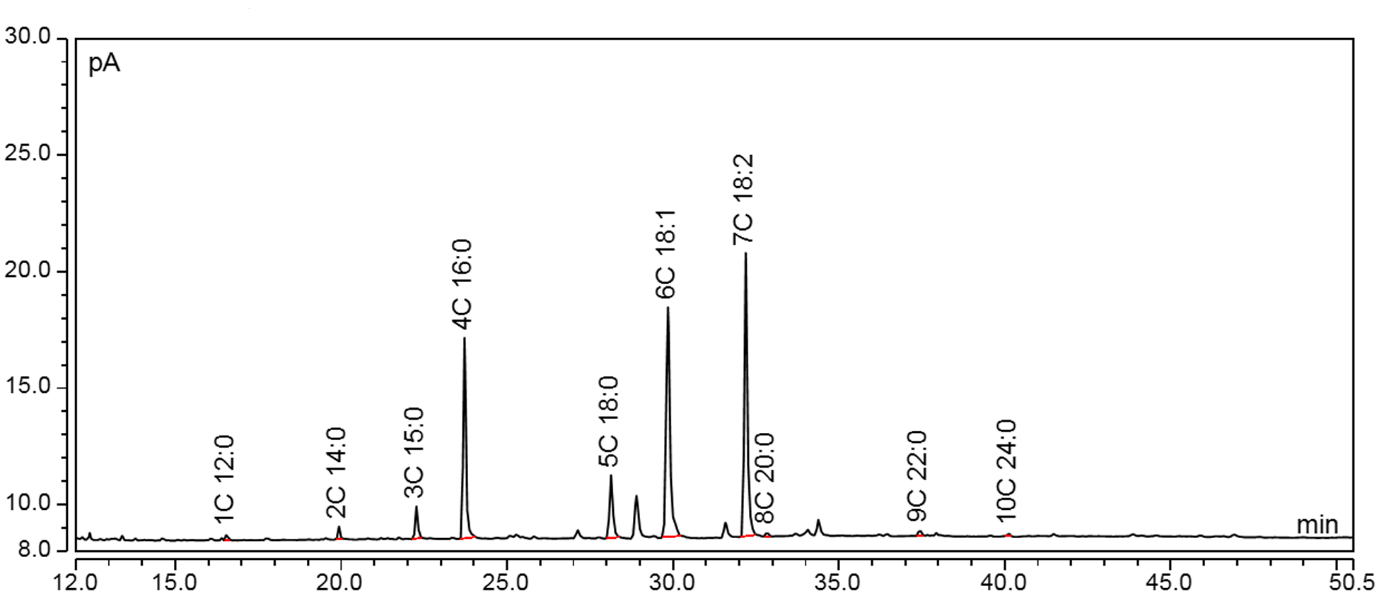


**S1 Fig.** Gas chromatography chromatogram for fatty acids present in the control diet.

**S1 Table. Gas chromatography analysis of different fatty acids present in the control diet.**

| **Sl. No.** | **Peak name** | **Compound name** | **Retention time (min)** | **Area pA min** | **Rel. Area %** |
| --- | --- | --- | --- | --- | --- |
| 1 | C 12:0 | Lauric acid | 16.550 | 0.019 | 0.47 |
| 2 | C 14:0 | Myristic acid | 19.925 | 0.040 | 1.00 |
| 3 | C 15:0 | Pentadecanoic acid | 22.267 | 0.131 | 3.25 |
| 4 | C 16:0 | Palmitic acid | 23.717 | 0.825 | 20.51 |
| 5 | C 18:0 | Stearic acid | 28.132 | 0.316 | 7.86 |
| 6 | C 18:1 | Oleic acid | 29.848 | 1.334 | 33.16 |
| 7 | C 18:2 | Linoleic acid | 32.190 | 1.302 | 32.37 |
| 8 | C 20:0 | Arachidic acid | 32.833 | 0.017 | 0.42 |
| 9 | C 22:0 | Behenic acid | 37.428 | 0.028 | 0.69 |
| 10 | C 24:0 | Lignoceric acid | 40.125 | 0.011 | 0.27 |
| **Total** | | | | **4.022** | **100.00** |


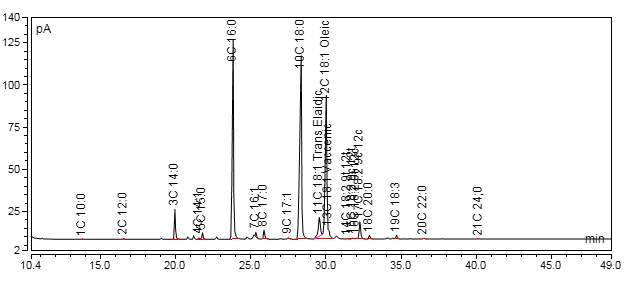


**S2 Fig.** Gas chromatography chromatogram for fatty acids in the high-fat diet.

**S2 Table. Gas chromatography analysis of different fatty acids present in the HF diet.**

| **Sl. No** | **Peak name** | **Compound name** | **Retention time (min)** | **Area pA min** | **Rel. Area %** |
| --- | --- | --- | --- | --- | --- |
| 1 | C 10:0 | Decanoic acid | 13.810 | 0.019 | 0.04 |
| 2 | C 12:0 | Lauric acid | 16.560 | 0.048 | 0.10 |
| 3 | C 14:0 | Myristic acid | 19.955 | 1.370 | 2.83 |
| 4 | C 14:1 | Myristoleic Acid | 21.563 | 0.085 | 0.18 |
| 5 | C 15:0 | Pentadecanoic Acid | 21.782 | 0.333 | 0.69 |
| 6 | C 16:0 | Palmitic acid | 23.827 | 12.391 | 25.55 |
| 7 | C 16:1 | Palmitoleic acid | 25.342 | 0.234 | 0.48 |
| 8 | C 17:0 | Margaric acid | 25.878 | 0.595 | 1.23 |
| 9 | C 17:1 | Heptadecanoic acid | 27.497 | 0.101 | 0.21 |
| 10 | C 18:0 | Stearic acid | 28.350 | 16.633 | 34.30 |
| 11 | C 18:1 | Trans Elaidic acid | 29.552 | 1.847 | 3.81 |
| 12 | C 18:1 | Oleic acid | 30.015 | 12.313 | 25.39 |
| 13 | C 18:1 | Vaccenic acid | 30.170 | 0.625 | 1.29 |
| 14 | C 18:2 9t 12t | Linolelaidic acid | 31.428 | 0.033 | 0.07 |
| 15 | C 18:2 9c 12t | Linoleic acid | 31.772 | 0.091 | 0.19 |
| 16 | C 18:2 9t 12c | Linoleic acid | 31.985 | 0.076 | 0.16 |
| 17 | C 18:2 9c 12c | Linoleic acid | 32.242 | 1.170 | 2.41 |
| 18 | C 20:0 | Arachidic acid | 32.888 | 0.237 | 0.49 |
| 19 | C 18:3 | Alpha-linolenic acid | 34.690 | 0.231 | 0.48 |
| 20 | C 22:0 | Behenic acid | 36.492 | 0.053 | 0.11 |
| 21 | C 24:0 | Lignoceric acid | 40.168 | 0.016 | 0.03 |
| **Total** | | | | **48.498** | **100.00** |

**References**

1. ISO. Animal and vegetable fats and oils: Gas chromatography of fatty acid methyl esters: Part 2: Preparation of methyl esters of fatty acids, International Organization for Standardization ISO 12966-2:2017(E), 3rd ed.; 2017.
2. Lisa SA, Alam MM, Bristy SA, Rahim A, Kabir MA. Qualitative enhancement and *trans*-fat depletion of different used frying oils with adsorbents, ACS Food Science & Technology 2024; 4(7): 1652-1661. DOI: 10.1021/acsfoodscitech.4c00110.
