# Supplementary figures and images for: Purple potato extract modulates fat metabolizing genes expression, prevents oxidative stress, hepatic steatosis, and attenuates high-fat diet-induced obesity in male rats
Source: PLoS One. 2025 Apr 1;20(4):e0318162. doi: 10.1371/journal.pone.0318162 (PMC11960900; doi:10.1371/journal.pone.0318162)

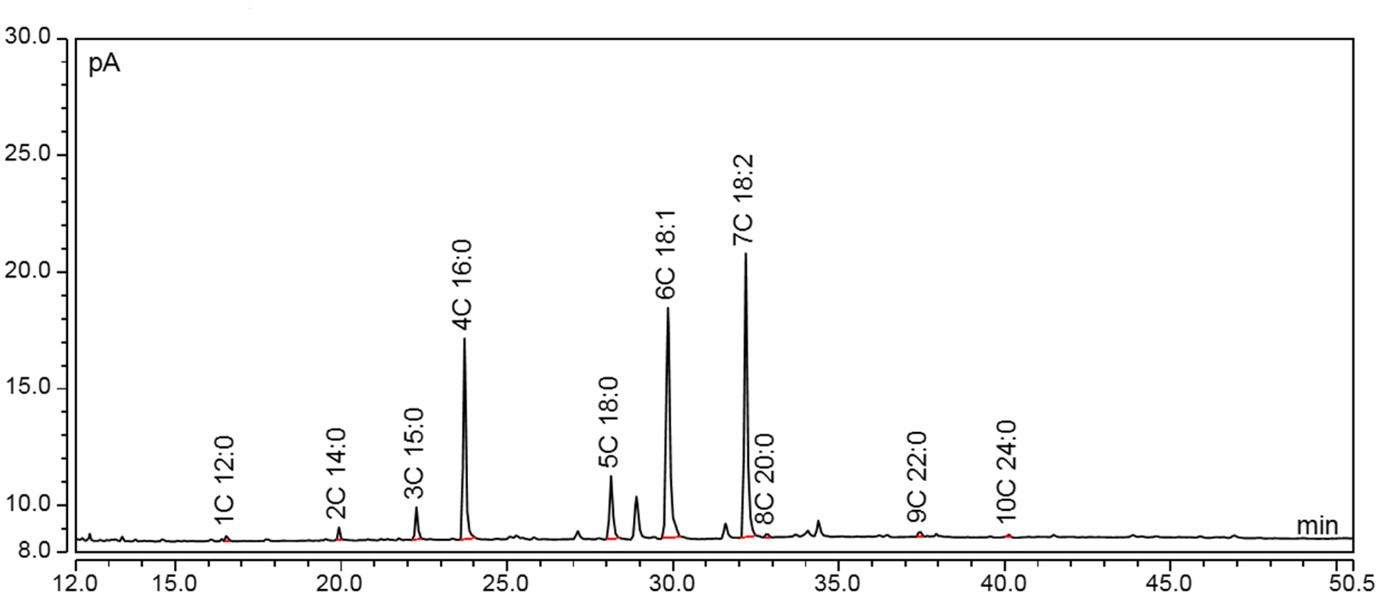

Supplement: S1 Fig — (JPG) [file pone.0318162.s002.jpg]

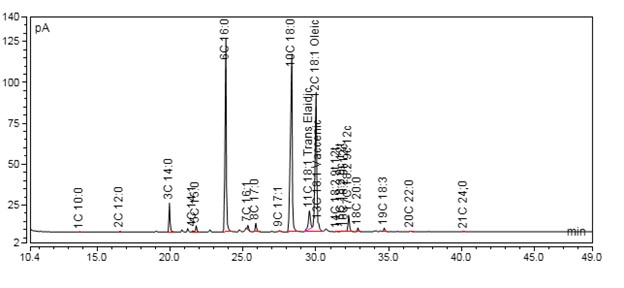

Supplement: S2 Fig — (JPG) [file pone.0318162.s003.jpg]
