# Supplementary material for: Purple potato extract modulates fat metabolizing genes expression, prevents oxidative stress, hepatic steatosis, and attenuates high-fat diet-induced obesity in male rats
Source: PLoS One. 2025 Apr 1;20(4):e0318162. doi: 10.1371/journal.pone.0318162 (PMC11960900; doi:10.1371/journal.pone.0318162)
Supplement: S1 Table — (DOCX) [file pone.0318162.s004.docx]

**S1 Table. Gas chromatography analysis of different fatty acids present in the control.**

| **Sl. No.** | **Peak name** | **Compound name** | **Retention time (min)** | **Area pA min** | **Rel. Area %** |
| --- | --- | --- | --- | --- | --- |
| 1 | C 12:0 | Lauric acid | 16.550 | 0.019 | 0.47 |
| 2 | C 14:0 | Myristic acid | 19.925 | 0.040 | 1.00 |
| 3 | C 15:0 | Pentadecanoic acid | 22.267 | 0.131 | 3.25 |
| 4 | C 16:0 | Palmitic acid | 23.717 | 0.825 | 20.51 |
| 5 | C 18:0 | Stearic acid | 28.132 | 0.316 | 7.86 |
| 6 | C 18:1 | Oleic acid | 29.848 | 1.334 | 33.16 |
| 7 | C 18:2 | Linoleic acid | 32.190 | 1.302 | 32.37 |
| 8 | C 20:0 | Arachidic acid | 32.833 | 0.017 | 0.42 |
| 9 | C 22:0 | Behenic acid | 37.428 | 0.028 | 0.69 |
| 10 | C 24:0 | Lignoceric acid | 40.125 | 0.011 | 0.27 |
| **Total** | | | | **4.022** | **100.00** |
