# Supplementary material for: Purple potato extract modulates fat metabolizing genes expression, prevents oxidative stress, hepatic steatosis, and attenuates high-fat diet-induced obesity in male rats
Source: PLoS One. 2025 Apr 1;20(4):e0318162. doi: 10.1371/journal.pone.0318162 (PMC11960900; doi:10.1371/journal.pone.0318162)
Supplement: S2 Table — (DOCX) [file pone.0318162.s005.docx]

**S2 Table. Gas chromatography analysis of different fatty acids present in the HF diet.**

| **Sl. No** | **Peak name** | **Compound name** | **Retention time (min)** | **Area pA min** | **Rel. Area %** |
| --- | --- | --- | --- | --- | --- |
| 1 | C 10:0 | Decanoic acid | 13.810 | 0.019 | 0.04 |
| 2 | C 12:0 | Lauric acid | 16.560 | 0.048 | 0.10 |
| 3 | C 14:0 | Myristic acid | 19.955 | 1.370 | 2.83 |
| 4 | C 14:1 | Myristoleic Acid | 21.563 | 0.085 | 0.18 |
| 5 | C 15:0 | Pentadecanoic Acid | 21.782 | 0.333 | 0.69 |
| 6 | C 16:0 | Palmitic acid | 23.827 | 12.391 | 25.55 |
| 7 | C 16:1 | Palmitoleic acid | 25.342 | 0.234 | 0.48 |
| 8 | C 17:0 | Margaric acid | 25.878 | 0.595 | 1.23 |
| 9 | C 17:1 | Heptadecanoic acid | 27.497 | 0.101 | 0.21 |
| 10 | C 18:0 | Stearic acid | 28.350 | 16.633 | 34.30 |
| 11 | C 18:1 | Trans Elaidic acid | 29.552 | 1.847 | 3.81 |
| 12 | C 18:1 | Oleic acid | 30.015 | 12.313 | 25.39 |
| 13 | C 18:1 | Vaccenic acid | 30.170 | 0.625 | 1.29 |
| 14 | C 18:2 9t 12t | Linolelaidic acid | 31.428 | 0.033 | 0.07 |
| 15 | C 18:2 9c 12t | Linoleic acid | 31.772 | 0.091 | 0.19 |
| 16 | C 18:2 9t 12c | Linoleic acid | 31.985 | 0.076 | 0.16 |
| 17 | C 18:2 9c 12c | Linoleic acid | 32.242 | 1.170 | 2.41 |
| 18 | C 20:0 | Arachidic acid | 32.888 | 0.237 | 0.49 |
| 19 | C 18:3 | Alpha-linolenic acid | 34.690 | 0.231 | 0.48 |
| 20 | C 22:0 | Behenic acid | 36.492 | 0.053 | 0.11 |
| 21 | C 24:0 | Lignoceric acid | 40.168 | 0.016 | 0.03 |
| **Total** | | | | **48.498** | **100.00** |
